# Supplementary material for: RET Functions as a Dual-Specificity Kinase that Requires Allosteric Inputs from Juxtamembrane Elements
Source: Cell Rep. 2016 Dec 22;17(12):3319–32. doi: 10.1016/j.celrep.2016.11.061 (PMC5199340; doi:10.1016/j.celrep.2016.11.061)
Supplement: Document S1. Figures S1–S6 [file mmc1.pdf]

Cell Reports, Volume *17*

Supplemental Information

RET Functions as a Dual-Specificity Kinase that Requires Allosteric Inputs from Juxtamembrane Elements

Iván Plaza-Menacho, Karin Barnouin, Rachael Barry, Annabel Borg, Mariam Orme, Rakhee Chauhan, Stephane Mouilleron, Rubén J. Martínez-Torres, Pascal Meier, and Neil Q. McDonald

Figure S1 Plaza-Menacho et al.

A B

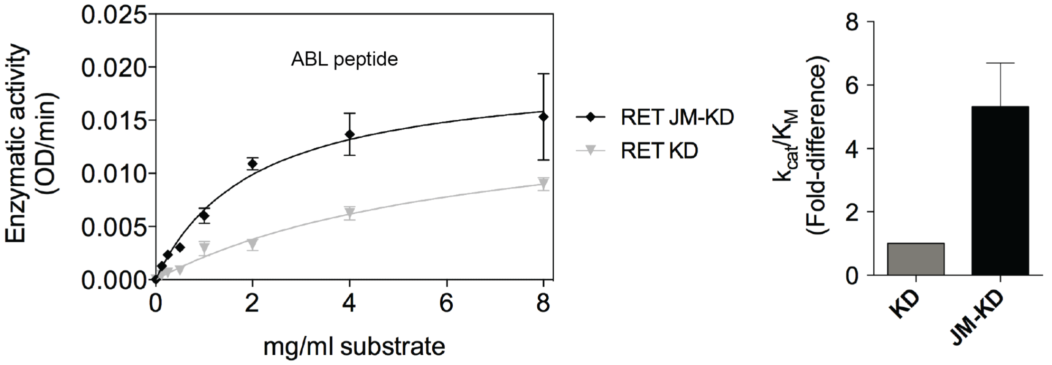

C

	Mw (kDa)	T_m (°C)	K_d (μM)	k_{cat} (min^{-1})	K_M (μM)	k_{cat}/K_M (min^{-1} μM^{-1})
**JM-KD**	41.6	41.0	37.5 ± 3	8.84 ± 0.03	305.0 ± 54	0.029
**KD**	33.9	42.2	64.3 ± 10	1.56 ± 0.15	269.6 ± 55	0.0058

D

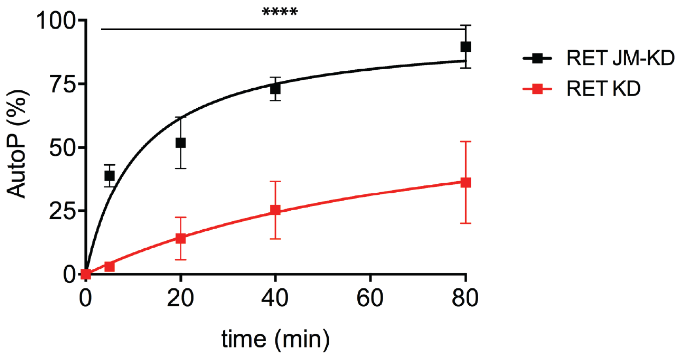

Figure S1 (related to Figure 1)

The juxtamembrane (JM) segment enhances RET catalytic domain activity.

(A-B) Enzymatic activity of RET JM-KD and RET KD measured against increasing concentrations of an exogenous ABL derived peptide substrate (sequence EAIYAAPFAKKK) showed increased catalytic efficiency (larger k_{cat}/K_M constant) towards the peptide-substrate. These data indicate increased RET catalytic activity promoted by the JM-region. Data represent mean ± standard error of the mean (SEM) from 2 experiments with 2-3 replicates each.

(C) Experimentally determined molecular weight (Mw), melting temperatures (T_m), dissociation constants (K_d) and enzyme kinetics (k_{cat}, K_M, k_{cat}/K_M for ATP) obtained by dynamic light scattering (DLS), ThermoFluor, ITC and enzymatic assays, respectively, as described in experimental procedures.

(D) Quantitation of WB data of figure 1B. Data represent the mean of phospho-tyrosine antibody (4G10) signal (i.e. autophosphorylation, percentage) normalized to total protein ± SEM, n=6. Statistics for RET JM-KD versus KD, **** $p<0.0001$ two-way Anova Bonferroni test.

Figure S2 Plaza-Menacho et al.

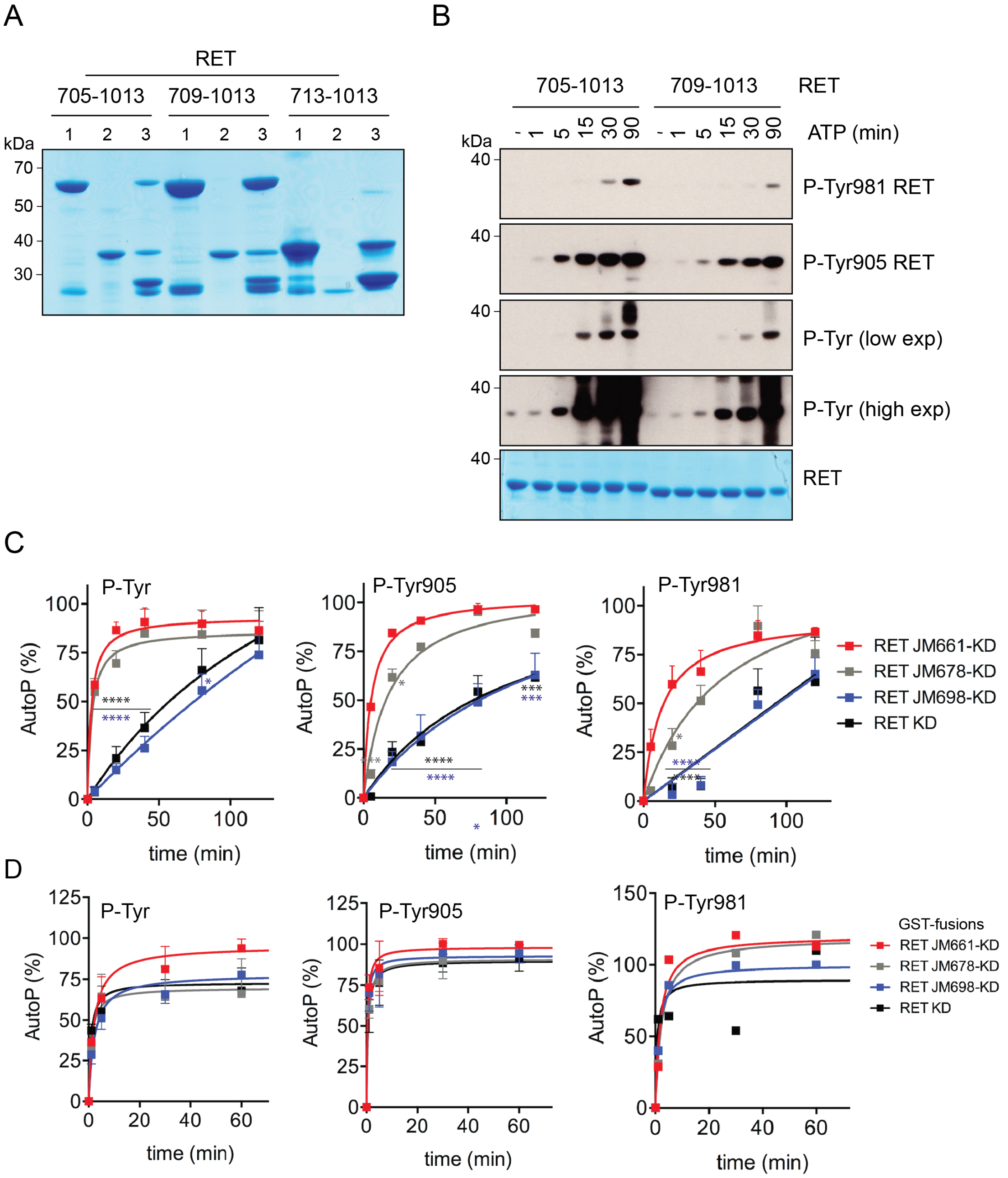

Figure S2 (related to figure 2)

Impact of fine deletions proximal to JM-segment and core kinase domain boundary and quantitation of figure 2 autophosphorylation assays.

Deletions targeting the transition from the JM-segment to the beginning of the RET catalytic core from residues 705 to 712 were evaluated in expression analysis and in phosphorylation assays.

(A) While recombinant RET catalytic domain starting at residue 709 was stable in solution, the construct starting from residue 713 gave rise to an unstable protein and

did not express properly. Lane 1 indicates GST-fusion protein in beads, lane 2 eluted protein after protease cleavage and lane 3 beads after cleavage.

(B) In phosphorylation assays the truncated RET starting at residue 709 displayed slower kinetics of autophosphorylation compared to RET KD starting at residue 705, as indicated by the different antibodies.

(C) Quantitation of WB data of figure 2C. Data represent the mean of autophosphorylation (percentage) ± SEM of the indicated antibodies normalized to total protein, n=3. Statistics * $p< 0.05$, *** $p<0.001$, **** $p<0.0001$ two- way Anova Bonferroni test versus control (WT).

(D) Quantitation of WB data of figure 2D. Data represent the mean of autophosphorylation (percentage) ± SEM of the indicated antibodies normalized to total protein, n=2.

Figure S3 Plaza-Menacho et al.

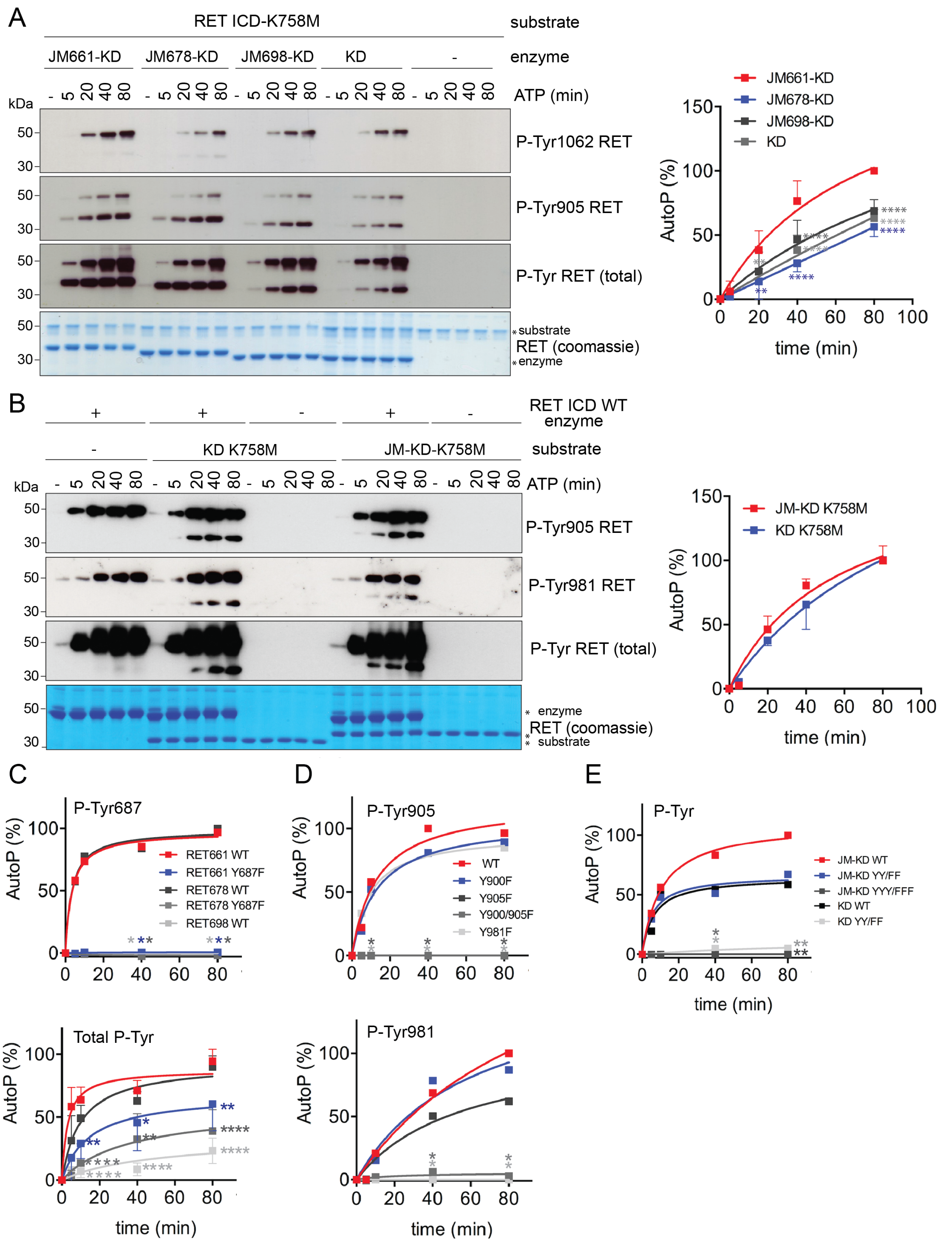

Figure S3 (related to figure 2 and 3)

Autophosphorylation rescue experiments in trans by RET JM-KD and RET KD and quantitation of RET autophosphorylation assays figure 3.

(A) To assess whether the JM-segment enhances the enzymatic properties of RET kinase, phosphorylation rescue experiments in trans using a catalytically impaired RET intracellular domain (ICD K758M) as a substrate were performed. Comparison between active RET JM-KD with different lengths of the JM-segment and RET KD core demonstrated an activating role for the JM terminal amino acid segment comprising residues 661-677. WB data quantitation of figure S3A, right panel. Data represent the mean of total phospho-tyrosine signal (i.e. autophosphorylation, percentage) normalized to total protein ± SEM, n=2. Statistics **** $p<0.0001$, ** $p<0.01$ two- way Anova Bonferroni test versus control (JM661-KD).

(B) To investigate whether the JM-region impacts on the substrate presentation properties of the receptor the reciprocal experiment was performed. Catalytically impaired RET K758M both JM-KD and KD core were used as substrates. Phospho-rescue experiments in trans by active RET ICD revealed that the JM-region does not affect the substrate properties of RET kinase, as no significant differences were observed between the two substrate variants. Note that enzyme variants used in upper panel (see lower bands) recapitulate data from in situ autophosphorylation assays (see figures 1 and 2). Quantitation of WB data of figure S3B, right panel. Data represent the mean of total phospho-tyrosine signal (i.e. autophosphorylation, percentage) normalized to total protein ± SEM, n=2.

(C) Quantitation of WB data of figure 3A. Data represent the mean of phospho-Tyr687 (upper panel, n=1) and total phospho-tyrosine signal (lower panel n=3) percentage normalized to total protein ± SEM. Statistics **** $p<0.0001$, ** $p<0.01$, * $p<0.5$, two- way Anova Bonferroni test versus control (JM661-KD).

(D) Quantitation of WB data of figure 3B. Data represent the mean of phospho-Tyr905 (upper panel) and phospho-Tyr981 (lower panel) normalized to total protein (n=1). Statistics **** $p<0.0001$, ** $p<0.01$, * $p<0.5$, two- way Anova Bonferroni test versus control (JM661-KD).

(E) Quantitation of WB data of figure 3C. Data represent the mean of total phospho-tyrosine antibody (4G10) signal (n=1) normalized to total protein. Statistics ** $p<0.01$, * $p<0.5$, two- way Anova Bonferroni test versus control (JM661-KD).

Figure S4 Plaza-Menacho et al.

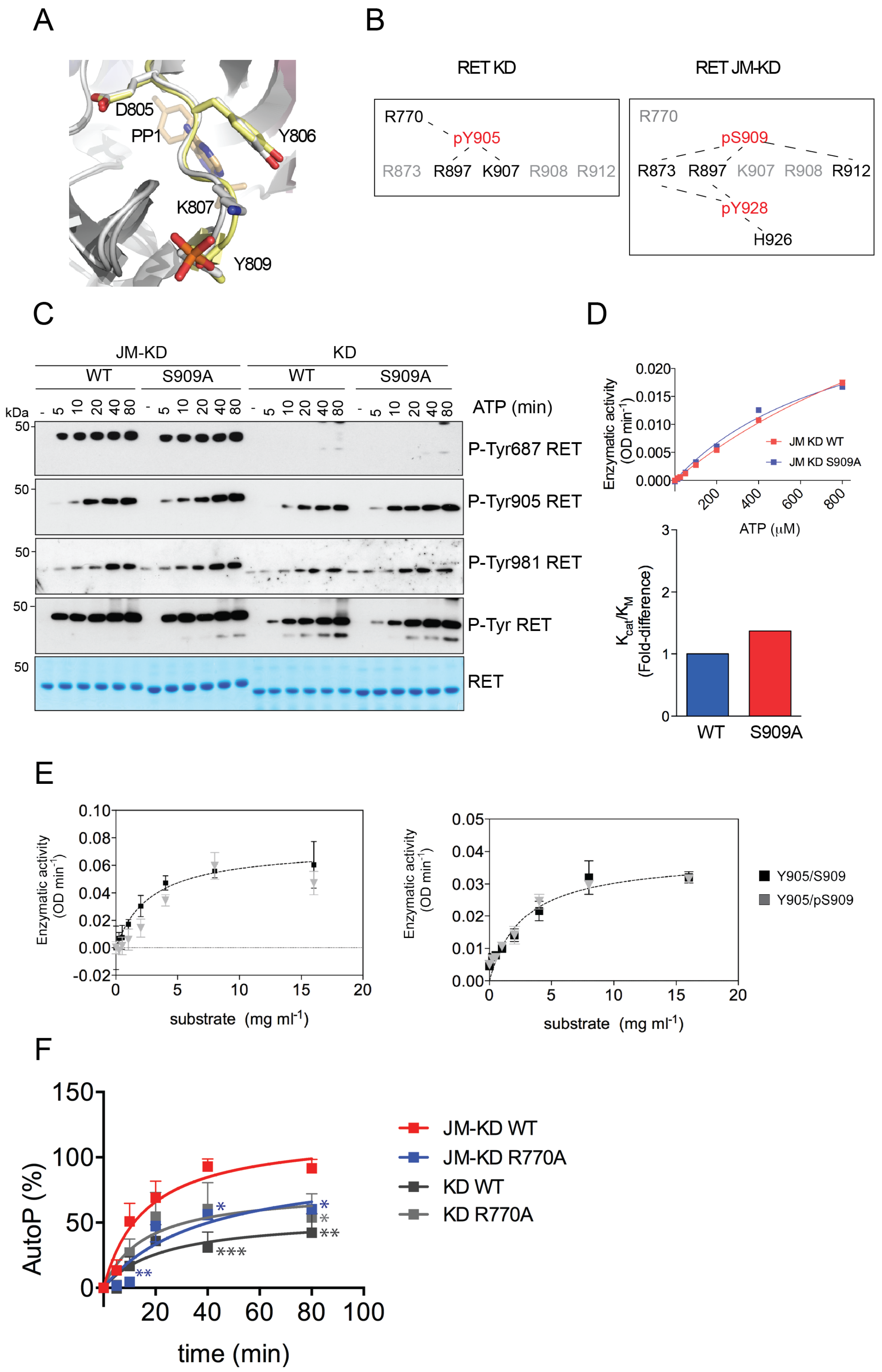

Figure S4 (related to figure 4)

Structural details of RET JM-KD crystal structure, functional evaluation of S909 on RET catalytic domain activity and quantitation of RET autophosphorylation assay figure 4E.

(A) Detailed view of the hinge region of RET JM-KD (PDB ID code 5FM3, yellow) superimposed onto the RET KD crystal structure (PDB ID code 2IVV, grey) showing no effect on the conformation of the kinase hinge by phospho-Y809.

(B) Schematic representation of basic residues engaged by either phospho-S909 and -Y928 from the JM-KD structure (PDB ID code 5FM2) or phospho-Y905 (PDB ID code 2IVV).

(C) WB analysis of in vitro time course autophosphorylation reaction using recombinant RET JM661-KD and RET KD WT and S909A mutants after addition of ATP (5 mM) and $MgCl_2$ (10 mM) for 0-80 minutes using the indicated antibodies.

(D) Enzymatic assay performed with recombinant purified (1µM) RET JM661-KD and RET KD WT and S909A mutants with increasing concentrations of ATP using an ABL derived peptide at a fixed concentration (4 mg/ml). Data represent mean ± SEM of n=3. Catalytic efficiency constants (k_{cat}/K_M, fold-difference) are shown on right panel.

(E) Phospho-S909 is not preferentially phosphorylated by RET catalytic domain in vitro. Enzyme kinetic experiment with recombinant purified phosphorylated-RET catalytic domain incubated with increasing concentrations of RET activation loop-derived peptides (with and without a phosphorylated S909 residue). Data shown are two independent experiments with different protein concentrations (5 µM left panel and 2.5 µM, right panel). These data is indicative of lack of intrinsic effect by phospho-S909 on RET catalytic activity.

(F) Quantitation of WB data of figure 4E. Data represent the mean of total autophosphorylation signal (percentage) ± SEM, n=3. Statistics *** $p<0.001$, ** $p< 0.01$, * $p< 0.05$, two- way Anova Bonferroni test versus control (JM-KD WT).

Figure S5 Plaza-Menacho et al.

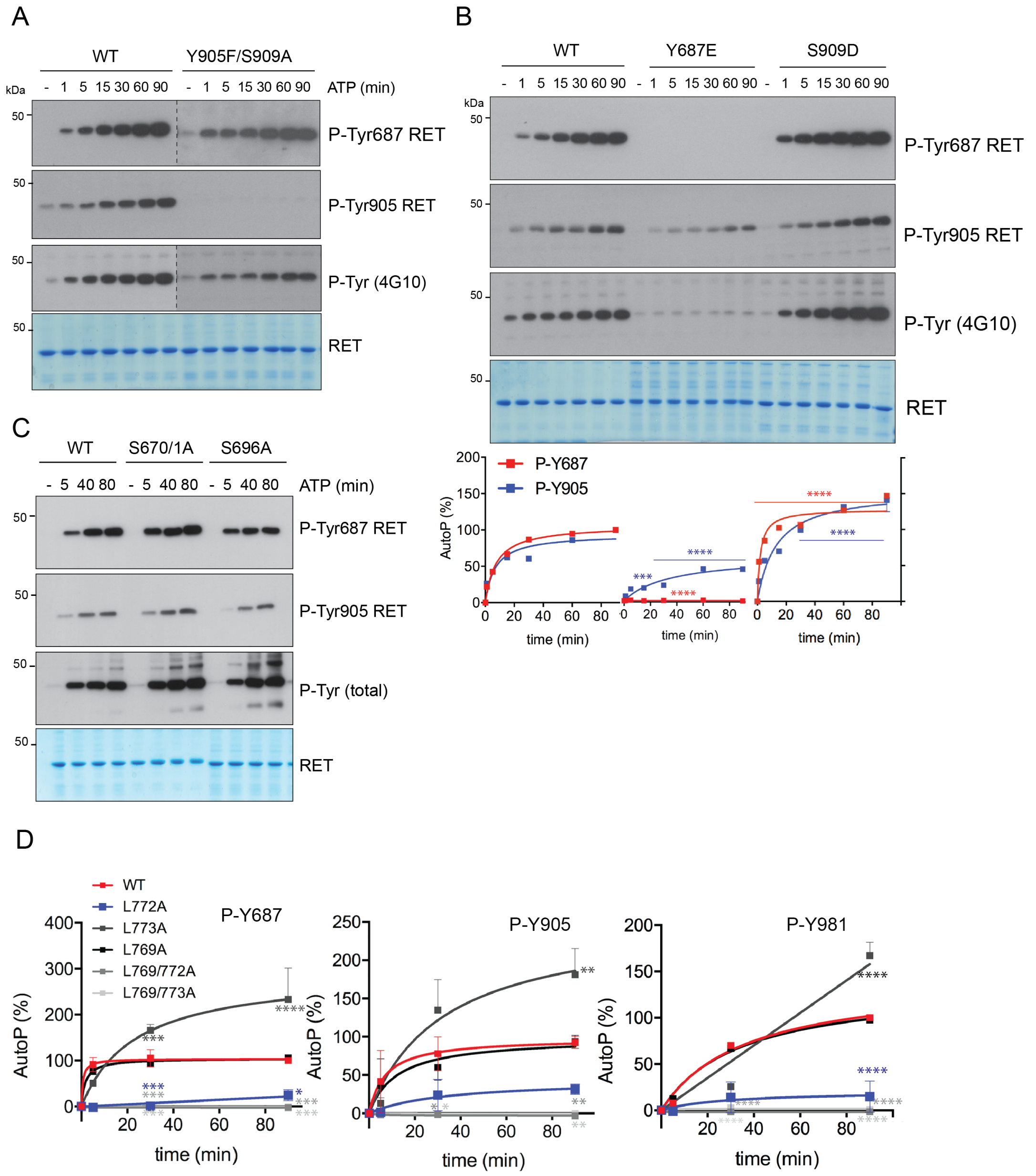

Figure S5 (related to figure 3, 4 and 5)

Functional characterization of distinct RET Tyr and Ser phospho-site mutants and quantitation of figure 5C autophosphorylation assay.

(A-C) WB analyses of recombinant RET JM-KD (1-2.5 µM) WT and indicated mutants incubated with saturating concentrations of ATP (5 mM) and $MgCl_2$ (10 mM) for 0-80/90 minutes using the indicated antibodies. Total amount of protein was assessed by Coomassie blue. Quantitation of WB data of figure S5B is depicted. Data represent the mean of autophosphorylation (percentage) ± SEM of the indicated antibodies, n=3. Statistics **** p<0.0001, *** p<0.001, two- way Anova Bonferroni test versus control (WT).

(D) Quantitation of WB data of figure 5C. Data represent the mean of autophosphorylation (percentage) ± SEM of the indicated antibodies, n=2. Statistics * p< 0.05, *** p<0.001, **** p<0.0001 two- way Anova Bonferroni test versus control (WT).

Figure S6   Plaza-Menacho et al.

A

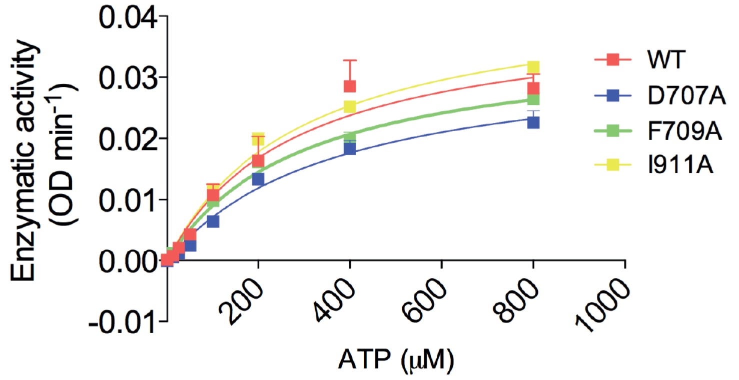

B

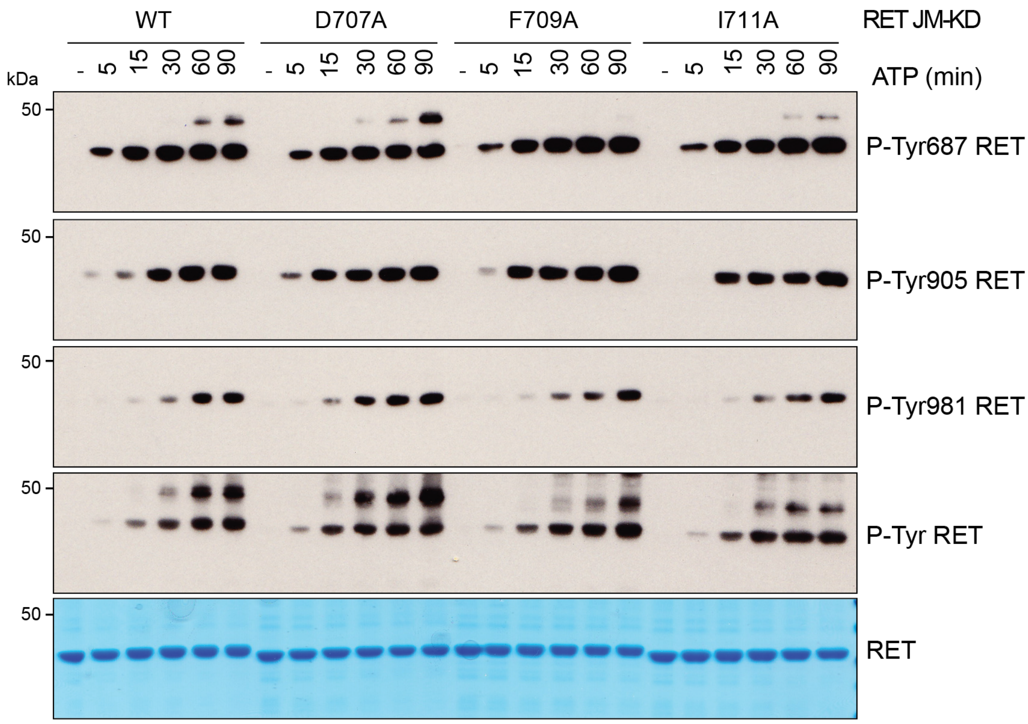

Figure S6 (related to figure 5 and 6)

Mutational analyses of RET DxFxI motif.

(A) Enzymatic assay performed with purified recombinant RET JM661-KD WT and indicated mutants (1 μM) incubated with increasing concentration of ATP using the ABL derived peptide EAIYAAPFAKKK at a constant 4 (mg/ml) concentration. Data represent mean ± SEM from 2 experiments with 6 replicates each, upper panel.

(B) WB analysis of recombinant purified RET samples from A (2.5 μM) stimulated with ATP (5 mM) and $MgCl_2$ (10 mM) for 0-80 minutes using the indicated antibodies, lower panel.
