# Supplementary material for: RET Functions as a Dual-Specificity Kinase that Requires Allosteric Inputs from Juxtamembrane Elements
Source: Cell Rep. 2016 Dec 22;17(12):3319–32. doi: 10.1016/j.celrep.2016.11.061 (PMC5199340; doi:10.1016/j.celrep.2016.11.061)
Supplement: Document S2. Article plus Supplemental Information [file mmc2.pdf]

Cell Reports

Article

RET Functions as a Dual-Specificity Kinase that Requires Allosteric Inputs from Juxtamembrane Elements

Graphical Abstract

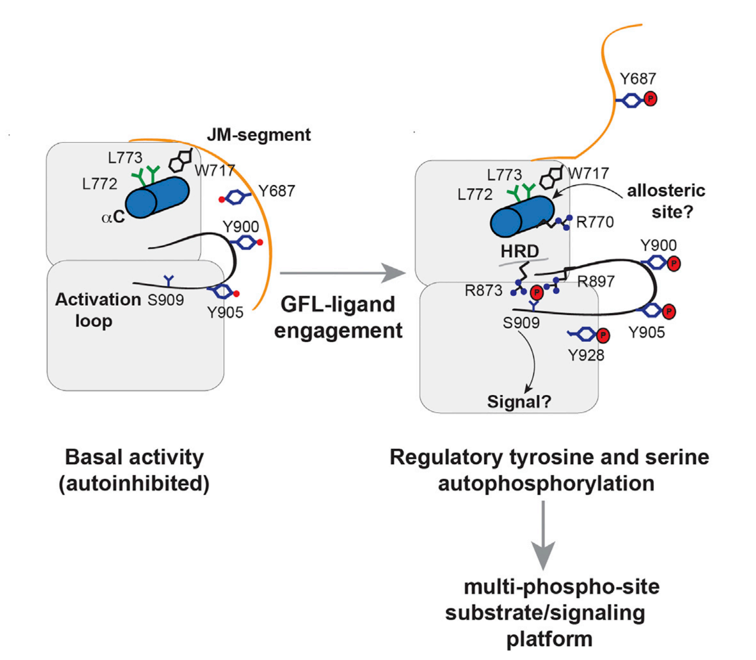

Authors

Iván Plaza-Menacho, Karin Barnouin, Rachael Barry, ..., Rubén J. Martínez-Torres, Pascal Meier, Neil Q. McDonald

Correspondence

ivan.plazamenacho@unibas.ch (I.P.-M.), neil.mcdonald@crick.ac.uk (N.Q.M.)

In Brief

Receptor tyrosine kinases exhibit a plethora of activation mechanisms despite highly homologous catalytic domains. Plaza-Menacho et al. find that RET tyrosine kinase activation and signaling require allosteric inputs from juxtamembrane elements as well as dual-specificity activity.

Highlights

- The JM segment enhances RET catalytic domain activity
- Structural visualization of activation-loop phospho-S909 engaging the HRD motif
- Phospho-S909 arises from an intrinsic RET dual-specificity kinase activity
- RET αC hydrophobic pocket is a potential drug-targetable allosteric site

Accession Numbers

5FM3
5FM2

CrossMark
Plaza-Menacho et al., 2016, Cell Reports *17*, 3319–3332
December 20, 2016 © 2016 The Author(s).
http://dx.doi.org/10.1016/j.celrep.2016.11.061

CellPress

Cell Reports
Article

OPEN ACCESS
CellPress

RET Functions as a Dual-Specificity Kinase that Requires Allosteric Inputs from Juxtamembrane Elements

Iván Plaza-Menacho,[1,7,8,*] Karin Barnouin,[2] Rachael Barry,[5] Annabel Borg,[3] Mariam Orme,[5] Rakhee Chauhan,[1] Stephane Mouilleron,[4] Rubén J. Martínez-Torres,[1] Pascal Meier,[5] and Neil Q. McDonald[1,6,*]
[1]Structural Biology Laboratory
[2]Protein Analysis and Proteomics
[3]Protein Production Facility
[4]Structural Biology Science Technology Platform
The Francis Crick Institute, 1 Midland Road, London NW1 1AT, UK
[5]The Breast Cancer Now Toby Robins Research Centre, Mary-Jean Mitchell Green Building, Institute of Cancer Research, SW3 6JB London, UK
[6]Department of Biological Sciences, Institute of Structural and Molecular Biology, Birkbeck College, Malet Street, WC1E 7HX London, UK
[7]Present address: Structural Biology and Biophysics, Biozentrum, University of Basel, Klingelbergtrasse 50/70, 4056 Basel, Switzerland
[8]Lead Contact
*Correspondence: ivan.plazamenacho@unibas.ch (I.P.-M.), neil.mcdonald@crick.ac.uk (N.Q.M.)
http://dx.doi.org/10.1016/j.celrep.2016.11.061

SUMMARY

Receptor tyrosine kinases exhibit a variety of activation mechanisms despite highly homologous catalytic domains. Such diversity arises through coupling of extracellular ligand-binding portions with highly variable intracellular sequences flanking the tyrosine kinase domain and specific patterns of autophosphorylation sites. Here, we show that the juxtamembrane (JM) segment enhances RET catalytic domain activity through Y687. This phospho-site is also required by the JM region to rescue an otherwise catalytically deficient RET activation-loop mutant lacking tyrosines. Structure-function analyses identified interactions between the JM hinge, αC helix, and an unconventional activation-loop serine phosphorylation site that engages the HRD motif and promotes phospho-tyrosine conformational accessibility and regulatory spine assembly. We demonstrate that this phospho-S909 arises from an intrinsic RET dual-specificity kinase activity and show that an equivalent serine is required for RET signaling in *Drosophila*. Our findings reveal dual-specificity and allosteric components for the mechanism of RET activation and signaling with direct implications for drug discovery.

INTRODUCTION

Vertebrates have close to 60 receptor tyrosine kinases (RTKs) that respond to a diverse set of extracellular polypeptide ligands by stimulating their intrinsic tyrosine kinase function. RTKs play key roles during embryogenesis and cellular homeostasis; they are also crucial at the origin and progression of many types of cancer (Lemmon and Schlessinger, 2010). Recent progress on the structural basis for EGFR, IR, and FGFR activation has emphasized the importance of RTK-specific or "private" mechanisms of activation for their catalytic domains involving flanking regions and asymmetrical and symmetrical arrangements of dimeric and higher-order oligomeric states (Bae and Schlessinger, 2010; Cabail et al., 2015; Jura et al., 2011; Lemmon et al., 2014). The activation mechanism operating in RET in these terms is currently unclear.

In the current RET paradigm for ligand-dependent RET activation, autophosphorylation (autoP) of several tyrosine residues within the cytoplasmic domain is required for cell signaling (Airaksinen et al., 1999; Plaza-Menacho et al., 2006). For other RTKs, such as the IR and FGFR2, ligand-dependent stimulation leads to kinase activation and phosphorylation of specific tyrosine residues, which relieve repressive *cis*-inhibitory interactions to enhance catalytic activity and to promote binding of phosphotyrosine-binding domain (PTB)- and Src homology 2 (SH2)-domain-containing proteins to transmit downstream signals (Chen et al., 2007; Hubbard, 1997). While the latter role for phosphorylation has been demonstrated for RET, its effect on catalytic activation has been only recently elucidated. In vitro, phosphorylation of the canonical RET activation loop has little effect on catalytic activity (Knowles et al., 2006; Plaza-Menacho et al., 2011). Indeed, RET activation-loop tyrosines Y900 and Y905 should not be considered activating, because they undergo delayed autoP and are not catalytically required (Plaza-Menacho et al., 2011, 2014a). A similar situation is found for the EGFR and non-RTK ACK1 (Lougheed et al., 2004; Zhang et al., 2006). In these cases, allosteric mechanisms have been identified to stimulate receptor activity independent of activation segment phosphorylation.

Cell-based studies have revealed the importance of the juxtamembrane (JM) segment in RET-receptor-mediated signaling, in particular Y687, a known phospho-tyrosine binding site for SHP2

This is an open access article under the CC BY license (http://creativecommons.org/licenses/by/4.0/).

(Perrinjaquet et al., 2010). In addition, phosphorylation at RET S696 by protein kinase A (PKA) has also been reported. Mutation of S696 affected the ability of RET to activate the small GTPase RAC1 and stimulate formation of cell lamellipodia (Fukuda et al., 2002). Homozygous knockin mice carrying this mutation lacked enteric neurons in the distal colon, resulting from a migration defect of enteric neural crest cells (Asai et al., 2006), indicating a physiological role for a PKA-RET functional crosstalk. However, structural and molecular information about allosteric mechanisms promoted by the JM region on RET kinase activity are lacking. Taking into account that the role of the JM segment of EGFR family members is distinct from that of typical RTKs because it enhances, rather than inhibits, the catalytic activity (Li et al., 2003; Thiel and Carpenter, 2007), the nature of this coupling between the JM segment and catalytic domain for RET has not been properly explored.

In this study, we define flanking elements and phospho-sites required for RET catalytic domain activation and signaling. We show that the JM segment functions to increase RET catalytic domain activity through Y687. Structure-function analyses revealed a crosstalk among the JM hinge, αC helix, and serine phosphorylated activation loop. We demonstrate that the previously unreported S909 phospho-site arises from a dual-specificity RET kinase activity, unique among RTKs. We show that an equivalent serine in *Drosophila* RET is required for signaling in vivo. Further structural and biochemical examination revealed an RET αC hydrophobic pocket as a potential drug-targetable allosteric site.

RESULTS

The JM Segment Increases RET Tyrosine Kinase Activity

To define the functional impact of the JM segment on RET tyrosine kinase activity, we used purified recombinant RET kinase domain (KD; residues 705–1013) and RET KD with the JM segment (JM-KD; residues 661–1012; see Figure 1A) and performed a series of biochemical experiments. First, we measured the enzymatic parameters of RET JM-KD and RET KD against an exogenous peptide (Figures S1A and S1B). RET JM-KD showed a 5-fold increased catalytic efficiency (k_{cat}/K_M constant) toward the substrate, indicating increased RET enzymatic activity promoted by the JM region. To support these results further, we performed in vitro time-course autoP assays using saturating concentrations of ATP (5 mM) and $MgCl_2$ (10 mM) for 0–80 min (Figures 1B, upper panel and S1D). Western blot (WB) analysis demonstrated increased kinetics and total phosphorylation by RET JM-KD, as indicated by levels of phospho-tyrosine 4G10 antibody. The temporal sequence of RET autoP was also evaluated by label-free quantitative mass spectrometry (LFQMS) following a previously described protocol (Plaza-Menacho et al., 2014a). LFQMS analysis identified tyrosine residues—Y687, Y826, Y900, and Y905—which upon RET catalytic activation were efficiently phosphorylated in a time-dependent fashion (Figure 1B, lower panel). Signal $\log_2$ ratios of phosphorylated peptides standardized to their non-phosphorylated counterparts were plotted relative to a zero time point (Figures 1C and 1D). As indicated by the kinetics of saturation, JM segment Y687 undergoes faster autoP than activation-loop Y900 and Y905. Furthermore, enhanced phosphorylation kinetics for Y900 and Y905 by RET JM-KD were observed compared with RET KD. In particular, a significant difference was observed in the kinetics of the double-phosphorylated activation-loop peptide. Examination of the total cumulative phosphorylation for each site demonstrated that fully phosphorylated RET JM-KD was achieved between 20 and 40 min compared with the 80–90 min required for RET KD (Figure 1B). Taken together, these data demonstrated that the JM segment increases RET catalytic activity presumably through an allosteric means. Contrary to the EGFR (Jura et al., 2009), the JM region did not promote the formation of RET dimers in solution at protein concentrations used in the biochemical assays as assessed by dynamic light scattering (DLS; Figure S1C). The JM segment had no appreciable impact on the stability of RET KD as reported by thermal shift experiments (Figure S1C). However, the apparent affinity for ATP measured by isothermal titration calorimetry (ITC) was affected by 2-fold (RET JM-KD K_d = 37.5 ± 3.1 μM, RET KD K_d = 64.3 ± 10 μM; Figure S1C). In line with these results, RET JM-KD also displayed increased enzyme kinetic parameters for ATP (Figures 1E and S1C).

Mapping JM Elements Required for RET Catalytic Activation

To map key residues within the RET JM region, we generated a series of deletions and performed biochemical analyses. First, enzymatic assays were performed using an ABL-derived peptide, used previously as a good surrogate substrate for RET. Comparison of the catalytic efficiency (k_{cat}/K_M) among the different RET JM-KD deletions demonstrated that full-length JM segment starting at residue 661 (JM661) was required to achieve maximal catalytic activity (Figures 2A and 2B). Time-course autoP assays (0–80 min) using RET phospho-specific antibodies were performed to validate the enzymatic assays and LFQMS data. More rapid and elevated phosphorylation levels were observed by RET JM661 as indicated by total phospho-tyrosine and phospho-specific RET Y905 and Y981 antibodies, respectively (Figures 2C and S2C). In this context, the shorter RET JM698 behaved similarly to RET KD, showing slower kinetics. Faster Y905 and Y981 autoP was observed by RET JM661 compared with RET JM678, and even more significantly with RET JM698 or RET KD. These results confirmed an increased RET catalytic activity because of the JM segment and implicate the region between residues 661 and 697. Further truncations targeting the transition toward the RET catalytic core, especially residues 705–712, were evaluated in expression analyses and in autoP assays. While the recombinant RET catalytic domain starting at residue 709 was stable in solution, the construct starting from residue 713 gave rise to an unstable protein (Figure S2A). The RET catalytic domain starting at residue 709 displayed slower kinetics of phosphorylation compared with that beginning at residue 705 (Figure S2B).

Recombinant glutathione *S*-transferase (GST)-RET KD fusions with different lengths of the JM segment were used to assess the impact of "forced" dimerization on RET catalytic activity in solution. GST-RET fusions displayed faster kinetics and increased levels of phosphorylation than the untagged proteins (Figures 2D and S2D). However, such enhanced kinetics were independent of the length of the JM segment. Although in principle

OPEN
ACCESS
CellPress

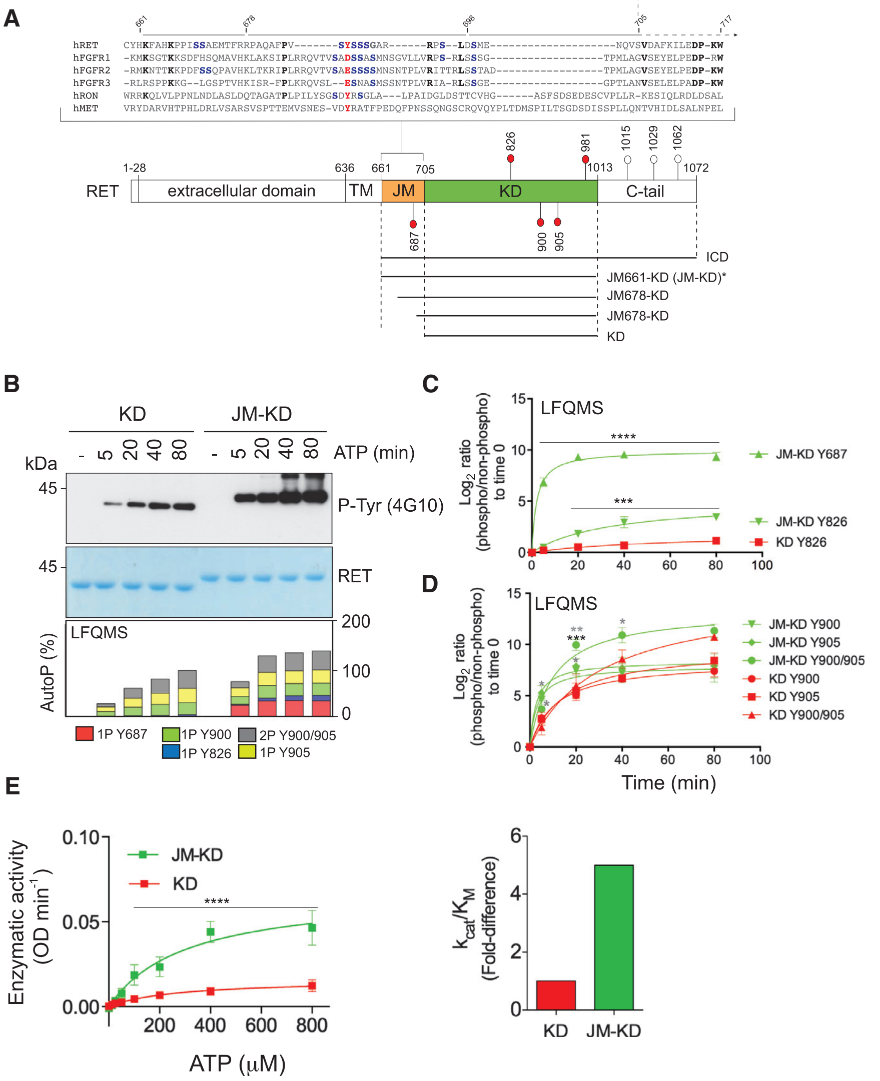

Figure 1. The JM Segment Enhances RET Catalytic Domain Activity In Vitro

(A) Alignment of selected JM sequences from human RTKs highlighting conserved residues in bold, serine residues in blue, and tyrosine in red. Selected acidic side chains at an equivalent position to Y687 of RET are also shown in red. Schematic diagram of discrete RET functional domains together with the phospho-sites (red spheres) analyzed in this study. White spheres correspond to sites outside the scope of this study. Residue numbering corresponds to human RET9 sequence (NP_065681.1). Dashed arrow depicts the transition from the JM segment to the RET catalytic core. Lower panel depicts RET constructs used in this study as indicated: RET intracellular domain (ICD; 661–1,072), RET kinase domain (KD; 705–1,013), and RET JM-KD variants starting at 661, 678, and 698, respectively. For crystallization purposes, an RET JM659-KD (659–1,013) construct was used in this study (*). Previously solved RET catalytic domain crystal structures used an RET KD (705–1,013) construct.

(B) Western blot (WB) analyses of purified recombinant RET JM-KD and RET KD (2.5 μM) treated with saturating concentrations of ATP (5 mM) and $MgCl_2$ (10 mM) for 0–80 min using the indicated antibody. Total amount of protein was assessed by Coomassie blue staining (upper panel). Lower panel shows a global time-dependent analysis by LFQMS (showing accumulative phosphorylation for each site) of the same samples. Data are representative of multiple independent experiments (n): n > 6 for WB and n = 3 for LFQMS.

(C and D) Phosphorylation kinetics of individual sites from (B) is shown. Data represent the mean $\log_2$ ratios of phosphorylated peptides normalized to their non-phosphorylated counterparts ± SEM, n = 3. Statistics for RET phospho-Y687, -Y826, -Y900, -Y905, and -Y900/Y905 (JM-KD versus KD): ****p < 0.0001, ***p = 0.009, two-way ANOVA Bonferroni test (black asterisks); *p < 0.05, **p < 0.005, multiple t test Sidak Bonferroni method (gray asterisks).

(E) Enzymatic assay performed with RET JM-KD and RET KD (1 μM) incubated with increasing concentrations of ATP at a fixed (4 mg/ml) ABL peptide concentration. Data represent the mean ± SEM, n = 4 from two different protein preparations; ****p < 0.0001, two-way ANOVA Bonferroni test (left panel). Catalytic efficiency constants (k_{cat}/K_M, fold difference) are depicted in the right panel.

this artificial system could lead to forced dimerization by the GST modules to dominate and override the effect of JM segment on RET activity, these data indicate that dimerization is not driving the increased activity promoted by the JM region in solution.

The JM Segment Increases RET Catalytic Activity without Affecting Substrate Presentation

The JM segment could potentially increase RET autoP by promoting a better substrate. To assess whether the JM segment also influences the substrate presentation properties of RET, we performed phosphorylation rescue experiments in *trans* using catalytically deficient RET K758M variants as substrates (Plaza-Menacho et al., 2014a). Consistent with earlier experiments, RET JM-KD-containing residues 661–677 were more active against catalytically deficient RET intracellular domain (ICD) K758M (i.e., substrate) than RET KD (Figure S3A). A reciprocal experiment was then performed using an active RET ICD against a catalytically deficient RET K758M in either JM-KD or KD context (Figure S3B). No significant differences were observed between the two substrate variants with or without the JM segment, indicating that the JM region makes RET kinase a better enzyme and not a better substrate for autoP.

JM Segment Y687 Promotes RET Catalytic Activity

The activating JM segment spanning residues 661–697 contains Y687, a known autoP site. To evaluate the functional role of this phospho-site in RET catalytic activity, we made Y687F mutant variants. AutoP assays of wild-type (WT) or Y687F mutants in a RET JM661 or JM678 context were compared with RET JM698 and showed a significant detrimental effect for Y687F

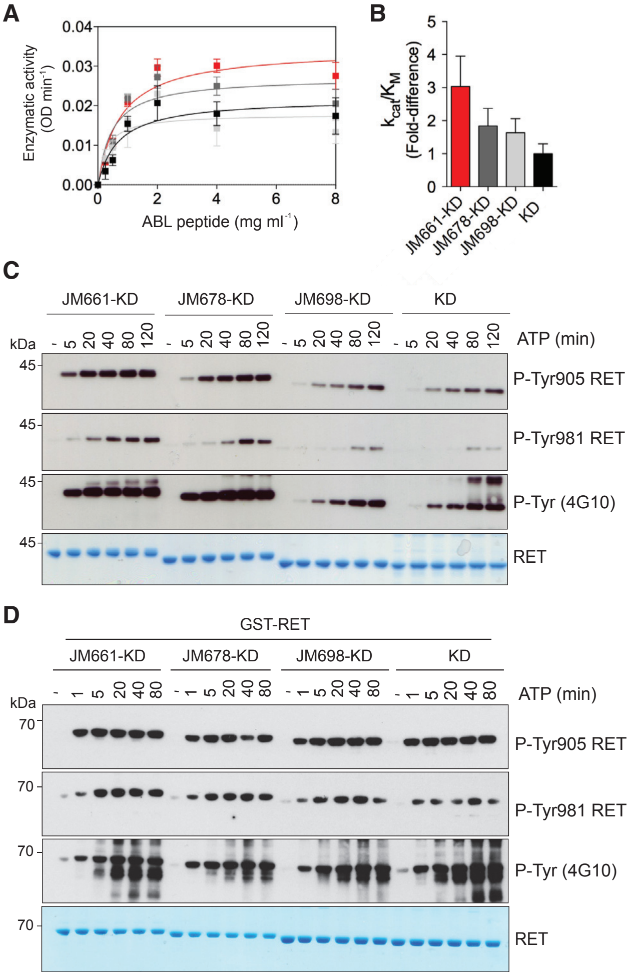

Figure 2. Mapping Key JM-Segment Elements Required for RET Catalytic Activation

(A) Enzymatic assay performed with purified recombinant (1 μM) RET JM-KD (with differing lengths) and RET KD varying the concentration of ABL peptide (sequence EAIYAAPFAKKK). Data are mean ± SEM and represent n = 3.

(B) Catalytic efficiency constants (k_{cat}/K_M, fold difference) from (A).

(C) WB analyses of purified recombinant RET JM-KD (2.5 μM, JM residues 661–705, RET JM661-KD), RET JM678-KD, RET JM698-KD, and RET KD treated with ATP (5 mM) and $MgCl_2$ (10 mM) for 0–120 min using the indicated antibodies.

(D) WB analyses of purified recombinant GST-RET fusions treated with ATP (5 mM) and $MgCl_2$ (10 mM) for 0–80 min using the indicated antibodies. Total amount of protein was assessed by Coomassie blue staining.

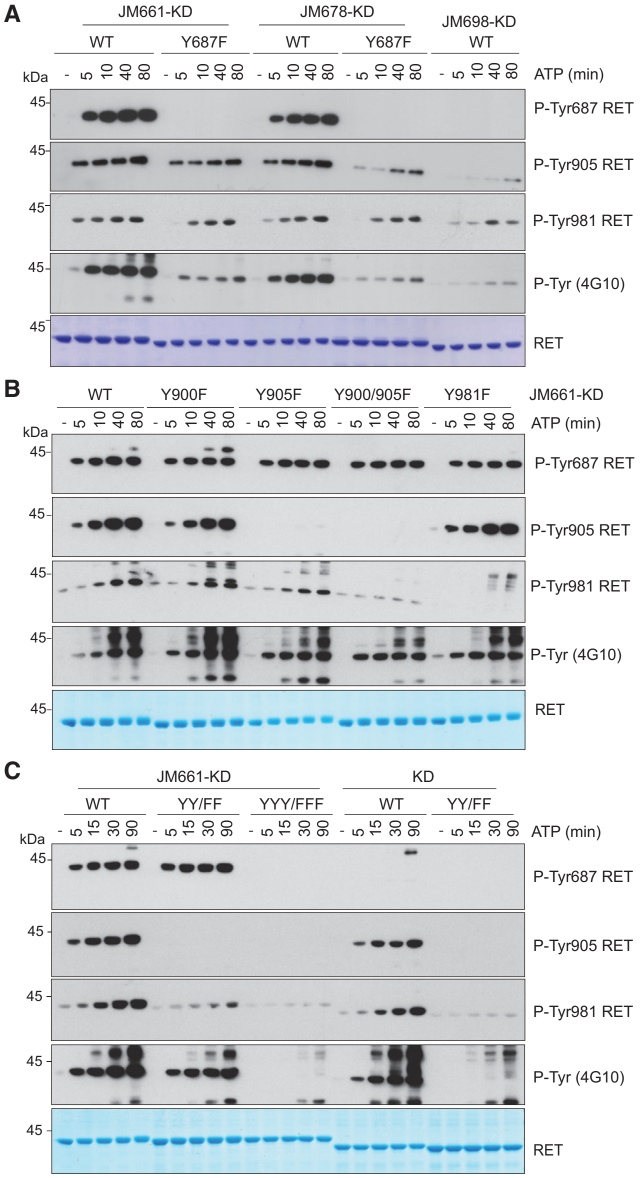

Figure 3. JM-Y687 Promotes RET Catalytic Activity and Rescues a Catalytically Deficient RET Activation-Loop Mutant Lacking Tyrosine

(A) WB analysis of purified recombinant RET JM661-KD, JM678-KD, and JM-698-KD (2.5 μM) WT and Y687F mutants stimulated with ATP (5 mM) and $MgCl_2$ (10 mM) for 0–80 min using the indicated antibodies.

(B) WB analysis of purified recombinant RET JM661-KD (2.5 μM) WT and indicated Y/F mutants (Y900F, Y905F, Y900/905F, Y981F) with ATP (5 mM) and $MgCl_2$ (10 mM) for 0–80 min using the indicated antibodies.

(C) WB analysis of purified recombinant RET JM661-KD and RET KD (2.5 μM) WT, Y900/905F (YY/FF), and Y687/900/905F (YYY/FFF) mutants as indicated stimulated with ATP (5 mM) and $MgCl_2$ (10 mM) for 0–90 min using the indicated antibodies. Total amount of protein was assessed by Coomassie blue staining.

mutants using both total phospho-tyrosine and phospho-specific RET Y905 and Y981 antibodies, respectively. In RET JM661, the effect of the Y687F mutant was reduced, suggesting 661–678 could partially compensate for the loss of Y687. Next, a phospho-specific polyclonal antibody was raised against a phospho-Y687 peptide. As expected, the phospho-specific Y687 antibody showed no signal for Y687F mutants nor RET JM698, but increased signal for WT RET JM661 compared with RET JM678 (Figures 3A and S3C). Previous data showed no impact of Y687 on RET ICD activity (Plaza-Menacho et al., 2014a). A dependency on Y687 is seen only in the absence of RET C-terminal (CT) sequences. One explanation would be if the JM and CT segments were in a spatially close proximity and could exhibit a compensatory effect masking a Y687 functional role (see Figure 5 and

Table 1. Data Collection and Refinement Statistics

	RET JM-KDd3	RETJM-KDd1
	5FM3	5FM2
Space group	P 6_3 2 2	P 6_3 2 2
Cell dimensions		
a, b, c, Å	98.5, 98.5, 146.3	98.4, 98.4, 144.5
α, β, γ	90.0°, 90.0°, 120.0°	90.0°, 90.0°, 120.0°
Resolution (outer resolution shell), Å	40 – 2.95 (3.11 – 2.95)	50 – 3.30 (3.48 – 3.30)
R_{sym} (%)	10.9 (79.5)	0.17 (0.89)
$R_{p.i.m.}$ (%)	4.1 (30.1)	0.05 (0.28)
I/σ	12.9 (2.8)	10.9 (2.8)
Completeness (%)	99.9 (100.0)	100 (100)
Redundancy	7.9 (8)	10.9 (11.3)
Resolution (outer resolution shell), Å	40 – 2.95 (3.37 – 2.95)	55.0 – 3.3 (3.75 – 3.3)
No. of unique reflections	9,360	6,686
R_{work}	19.9 (25.0)	23.4 (25.7)
R_{free}[a]	22.4 (28.9)	25.3 (29.4)
No. of atoms	2,151	2,105
Wilson B factor	76.8	79.4
Average isotropic *B* factors, Å^2	74.4	75.9
Rmsds		
Bonds, Å	0.002	0.002
Angles, °	0.66	0.65
Ramachandran plot (favored/allowed/disallowed), %	94.9/4.7/0.4	96.3/3.7/0.0

[a]A total of 5% of the data were set aside to compute R_{free}.

Discussion). Next, we assessed the effect of single Y900F, Y905F, and Y981F and double Y900/905F mutants on RET JM-KD activity. In contrast with the detrimental effect observed for Y687F mutants, replacement of the other phospho-sites (Y/F) did not disrupt RET autoP (Figures 3B and S3D). Of note, double activation-loop RET JM-KD Y900/905F mutant showed significant lower levels of Y981 phosphorylation despite no effect on total phosphorylation. More importantly, RET JM661-KD Y900/905F showed WT levels of phospho-Y687 and total phosphorylated RET kinase, indicating the JM segment is able to rescue the activity in *cis* of the catalytically deficient RET KD Y900/905F mutant (Plaza-Menacho et al., 2011, 2014a). These data demonstrate that crosstalk (i.e., rescue) between the JM segment and the activation-loop is required for RET catalytic function. Further evidence of coupling between the JM and activating segments was obtained by testing a triple RET JM-KD Y687F/Y900/905F mutant for tyrosine kinase activity (Figures 3C and S3E). Crucially, RET JM-KD Y687F was not able to rescue the catalytically deficient Y900/905F mutation. Altogether, these data demonstrate that Y687 is required for a proper allosteric input by the JM segment on RET catalytic activity able to overcome and stabilize a Y900/905F-deficient activation-loop mutant.

Crystallographic Identification of an Unexpected Activation-Loop Phospho-S909

We have determined two similar crystal structures of a construct containing the RET JM region and KD (amino acids 659–1013) at 3.3 and 2.95 Å, respectively (Table 1). Both crystal structures contained the PP1 tyrosine kinase inhibitor in the nucleotide-binding pocket, had an ordered proximal portion of the RET JM segment, and had a hyper-phosphorylated status with four sites phosphorylated (Y809, Y905, S909, and Y928) (Figures 4A and 4B). The structures differ slightly in resolution and in the occupancy of the phospho-S909 site. The enhanced multi-site phosphorylation status was surprising when compared with previously solved crystal structures of mono-phosphorylated RET catalytic domain (see PDB: 2IVT, 2IVU, 2IVV, and 4CKI), but consistent with biochemical data, indicating higher RET JM-KD levels of tyrosine kinase activity compared with RET KD (Figures 1 and 2). Residue Y809 is located within the RET hinge connecting the N-lobe and C-lobe of the RET KD, whereas Y905 and S909 are within the RET activation loop, and Y928 follows the WMAIE motif at the end of the activation segment between helixes α4 and α5 (Hanks et al., 1988). The presence of these phosphorylation sites impacts mainly on the activation-loop conformation detaching it from the body of the catalytic core without affecting the conformation of the hinge, as described later (Figures 4A, 4B, and S4A). Previously solved phosphorylated RET KD crystal structures (PDB: 2IVT, 2IVV, and 2IVU) showed phospho-Y905 tethers several basic side chains including R770 from the αC helix and residues R897 and K907 from the activation loop. In the crystal structures presented in this study, phospho-S909 displaces phospho-Y905 and adopts an approximate equivalent position by engaging activation segment residues R897 and R912, as well as R873 from the HRD motif instead (Figures 4B, 4C, and S4B). In this situation, Y905 does not engage the side chain of the αC helix R770; as a consequence, phospho-Y905 projects away from the body of the RET kinase to mimic a fully solvent-accessible conformer. The second unexpected phosphorylation site at Y928 is positioned beneath the tethered phospho-S909 and is likely to further disrupt interactions of phospho-Y905 with the activation loop. Phospho-Y928 forms hydrogen bonds with side chains of R873 (HRD motif) and activation loop R897 at the top and with H926 from beneath. Its partially buried position indicates the activation loop must have adopted an accessible conformation to fully expose Y928 to undergo phosphorylation. These data are consistent with a recent study where we showed enhanced substrate presentation in *trans* (i.e., activation-loop out conformer) in solution by an oncogenic RET M918T mutant targeting the P+1 substrate-binding pocket (Plaza-Menacho et al., 2014a).

Phospho-S909 Arises from an Intrinsic RET Dual-Specificity Kinase Activity

Full-length RET and RET ICD are known to be serine phosphorylated in cells and in vitro, respectively (Plaza-Menacho et al., 2014a; Takahashi et al., 1993). S909 is invariant in all RET sequences and is found only within a minority of RTKs in the human kinome (e.g., FGFR4, ROR1, and HER3). We did not detect S909 phosphorylation by mass spectrometry; however, when we used a specific antibody against an RET phospho-S909 epitope (pSQG), weak basal phospho-serine activity was observed for

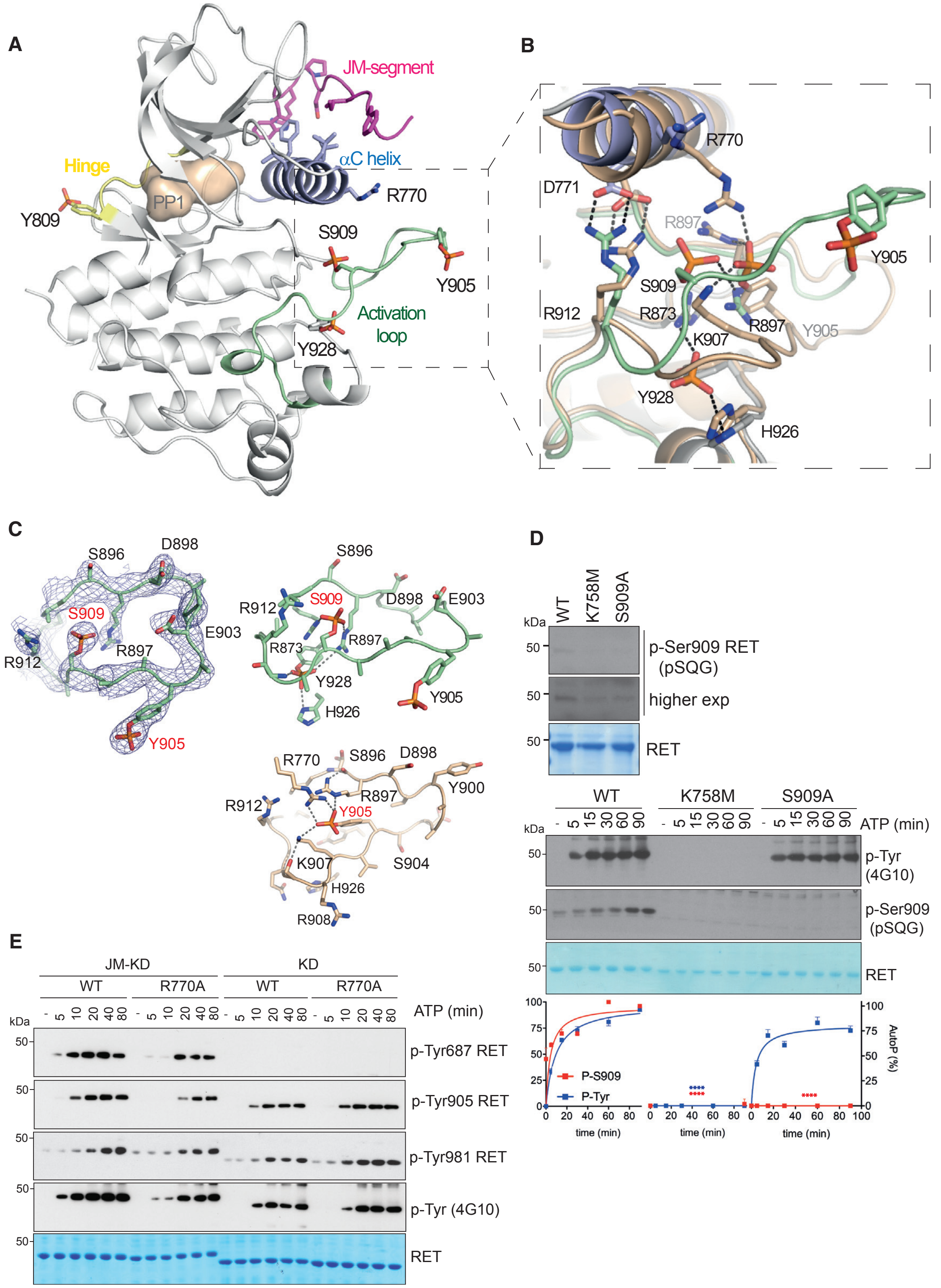

Figure 4. Crystallographic Identification of an Unexpected Activation-Loop Phospho-S909 Reveals Intrinsic RET Dual-Specificity Activity

(A) Cartoon representation of phosphorylated RET JM-KD structure bound to PP1 inhibitor. Selected residues (including phosphorylated side chains) and secondary structure elements are depicted with discrete colors: JM-segment residues D707 to K716 (magenta), αC helix (purple), hinge residues (yellow), and activation segment residues (green) are shown.

(B) Close-up of the activation-loop conformation and side chains in (A) (green) superposed with RET KD (tint wheat, PDB: 2IVV).

(legend continued on next page)

RET WT (Figure 4D, upper panel). RET S909 phosphorylation was dependent on the catalytic status of the receptor as indicated by lack of signal of a kinase-dead K758M mutant and was highly specific for S909 (i.e., no signal by a S909A mutant). These data suggested that, contrary to a constitutive phosphorylation event in *trans* on S909 by an unknown serine-threonine kinase as we initially hypothesized, RET could be a dual-specificity kinase that can autophosphorylate on S909. To test this hypothesis, we performed time-course autoP assays with RET ICD WT versus K758M and S909A mutants (Figure 4D, lower panel). As predicted from previous results, RET K758M showed no tyrosine kinase activity compared with RET WT and S909A mutant, which showed similar time-dependent tyrosine autoP (Figure S4C). Crucially, when a phospho-specific RET S909 epitope antibody was used, a time-dependent effect was seen only for RET WT, which showed phospho-serine levels saturating at 60–90 min after stimulation. In contrast, no signal was seen in the case of RET K758M or S909A mutants. Taken together, these data demonstrated that phospho-S909 arises from an intrinsic RET dual-specificity kinase activity not previously reported for an RTK.

Structure-Function Validation of RET JM-KD Crystal Structure

To interpret the increased JM-KD kinase activity from the new structure, we considered whether αC R770 side chain, which coordinates phospho-Y905 in the RET KD structure, could instead make contacts with the JM segment, thereby stabilizing a more active conformer independently of phospho-Y905. Assessing the functional impact of an R770A mutant in the context of both RET JM-KD and RET KD, we found the mutant was selectively impairing RET JM-KD activity but had not a measureable detrimental effect on RET KD (Figures 4E and S4F). These data implicate R770 in engaging the JM segment to increase RET catalytic domain activity. Second, we evaluated whether S909 was required for RET tyrosine kinase activity in vitro. Surprisingly, we did not observe any significant effect of the S909A mutant on RET activity in either enzyme kinetics using peptide substrates or in autoP assays (Figures S4C and S4D). Further enzymatic experiments using purified recombinant RET KD with increasing concentrations of activation-loop-derived S909 phospho- and non-phospho-peptides confirmed these results further (Figure S4E). These data indicate that analogously to RET KD tyrosine autoP sites, S909 is not intrinsically required for catalytic activity. We also considered that redundancy of phospho-S909 with phospho-Y905 could mask such a critical role. The latter possibility was further excluded by the lack of any functional effect observed by an RET JM-KD Y905F/S909A double mutant (Figure S5A). An equally plausible explanation is that multi-site phosphorylation of the RET activation segment could play a role in releasing phospho-Y905 or even phospho-S909 acting as a docking or adaptor site required for downstream signaling (see Discussion).

Structural Identification and Functional Validation of RET αC Hydrophobic Patch

The JM-KD structure revealed contacts from a short segment of the proximal JM region (residues D707 to W717) with a hydrophobic patch composed of residues from different structural elements including β4 (L790), β5 (L800, L801, L802), and αC helix (L769, L772, L773, F776, L779) (Figures 5A and 5B). This αC hydrophobic patch is present in many tyrosine and serine-threonine kinases and is frequently a site of regulation to assemble a functional regulatory (R) spine (Kannan et al., 2007; Kovacs et al., 2015; Thompson et al., 2009). Intramolecular contacts with this hydrophobic patch arise from interaction with either N- or C-terminal sequences flanking the KD (Jura et al., 2011). We noticed a passing similarity between RET αC hydrophobic patch-JM-segment interaction and the PIF pocket-hydrophobic motif interaction found in AGC kinases first described for the PDK1 serine-threonine kinase (where PIF is defined as the PDK1-interacting fragment) and PKA (Kannan et al., 2007; Biondi et al., 2000). Superposition of the RET JM-KD crystal structure with the PKA catalytic domain (PDB: 1ATP) suggests an equivalence between residues of the PKA hydrophobic motif located at its C terminus to contact the αC helix with those observed in the RET JM segment that engage the hydrophobic αC patch (Figures 5A and 5B). Our interest in this similarity was stimulated by the development of selective drugs against the PDK1 PIF pocket, suggesting the potential for targeting the same region of RET by chemical inhibitors as an alternative route to RET nucleotide pocket inhibition.

To biochemically probe the role of this αC hydrophobic patch on RET tyrosine kinase activity in vitro, we engineered individual mutants L769A, L772A, and L773A and double mutants L769/772A and L769/773A, and assessed the effect on autoP and enzyme kinetic assays (Figures 5C and 5D). Out of the three single-point mutants, L772A had a profound detrimental effect compared with WT and L769A, whereas L773A had a marked gain-of-function effect on RET kinase activity. The proximity of L772 and L773 on the αC helix and their opposing effects suggests a subtle conformational alteration of αC would be important for R-spine assembly and hence RET activation. We note that the insulin receptor kinase L1045 (structural equivalent to L773 of RET) directly contacts JM segment Y984 side chain stabilizing an auto-inhibited form (Li et al., 2003). By analogy, L773 could also potentially engage Y687 bound in a similar manner; this would explain why a L773A mutant stimulates RET activity (see Discussion). In contrast, L772A gave rise to a loss-of-function effect. We hypothesize that an RET L772A mutant would not create a constitutively active RET (based on

(C) The 2Fo-Fc electron density map of phosphorylated activation-loop from PDB: 5FM2 is shown as blue mesh countered at 1σ. Cartoon representation of basic residues engaged by either phospho-S909/Y928 from the JM-KD structure (upper panel, PDB: 5FM2) or phospho-Y905 (from PDB: 2IVV).

(D) WB analyses using a specific antibody against RET phospho-S909 epitope (pSQG) using recombinant RET ICD WT, K758M, and S909A (upper panel) and in vitro time-course autoP assay in the presence of ATP (5 mM) and $MgCl_2$ (10 mM) for 0–90 min (lower panel). Total RET protein was evaluated by Coomassie blue staining. Quantitation of WB data of Figure 4D is depicted. Data represent the mean of autoP (percentage) normalized to total protein ± SEM of the indicated antibodies, n = 3. Statistics: ****$p < 0.0001$, two-way ANOVA Bonferroni test versus control (WT).

(E) WB analysis of in vitro time-course autoP assay using RET JM661-KD and RET KD core wild-type (WT) and R770A mutants after adding ATP (5 mM) and $MgCl_2$ (10 mM) for 0–80 min using the indicated antibodies.

OPEN
ACCESS
CellPress

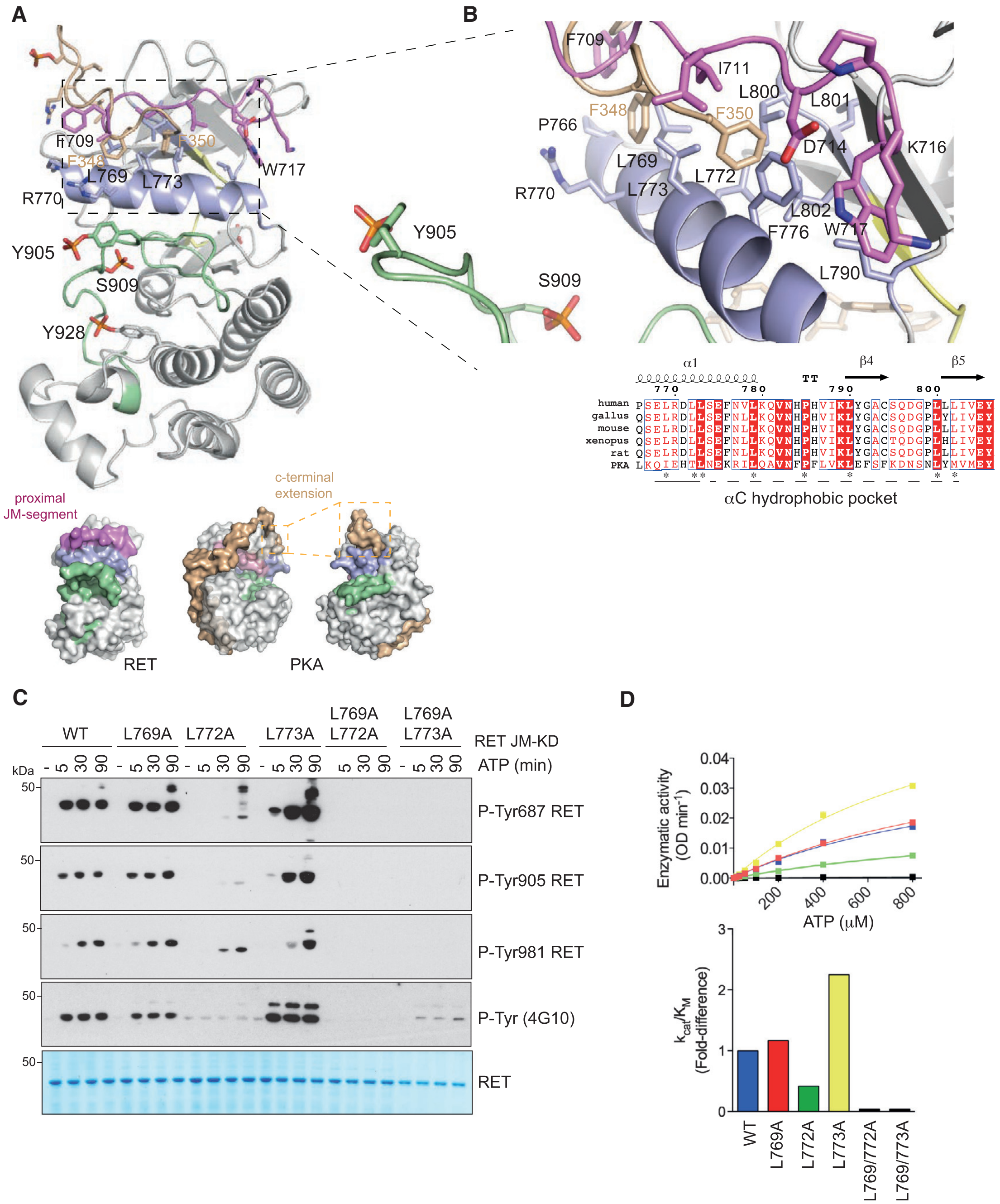

Figure 5. Structure-Function Analysis of RET αC Hydrophobic Patch

(A) Upper panel shows a cartoon representation of RET JM-KD structure, colored according to Figure 4A, with the superposition of PKA C-terminal residues (PDB: 1ATP). Lower panel shows two views of a surface representation of PKA catalytic subunit together with one of the RET JM-KD structures. The PKA C-terminal segment (pale brown), αC helix (purple), and activation loop (green) are depicted together with selected residues.

(B) Close-up of a superposition of RET αC hydrophobic patch contact residues from the proximal JM-residues (D707 to W717) together with the C-terminal hydrophobic motif (FTDF) from PKA. Selected residues and secondary structural elements are depicted as in (A); some residues have been omitted for clarity. Alignment of RET sequences from different species indicating secondary structural elements and key residues (*) implicated in the αC hydrophobic patch (lower panel).

(C) WB analysis of in vitro time-course phosphorylation assay using RET JM661-KD WT and indicated mutants after addition of ATP (5 mM) and $MgCl_2$ (10 mM) for 0–80 min using the indicated antibodies.

(D) Enzymatic assay performed with recombinant purified (1 μM) RET JM661-KD WT and indicated mutants with increasing concentrations of ATP using the ABL peptide at a fixed concentration (4 mg/ml). The corresponding fold-difference in k_{cat}/K_M values is shown in the lower panel. Data represent mean ± SEM, n = 2.

the BRAF paradigm, see Discussion), but would rather impact on the catalytically required K758-E775-D892 tether and would therefore have a similar impact to the loss-of-function K758M mutant. Double mutants L769/772A and L769/773A were both impaired in their catalytic activity. The contribution of residues from the proximal JM region (i.e., D707, F709, and I711) was also evaluated, revealing lack of functional effect (Figures S6A and S6B). These data suggest that other N-terminal residues from the JM segment not captured in the crystal structure may be relevant for this interaction (e.g., Y687). Alternatively, residues proximal to the transition between JM segment and catalytic domain boundary could also be implicated (see RET W717 Contributes to the Assembly of the JM Hinge and R-Spine and Discussion sections). These data implicate residues L772 and L773 from the αC helix as key determinants in achieving an active conformer and may potentially, by analogy to PDK1, provide an alternative druggable pocket to target within RET.

RET W717 Contributes to the Assembly of the JM Hinge and R-Spine

Further structural examination of the αC hydrophobic patch highlighted W717, a highly conserved residue preceding the β-1 strand in many protein kinases including SRC, BTK, EGFR, and BRAF that separates the JM segment from the core catalytic domain (Figure 1A). In the case of BRAF (W342), this residue is important for capping the R-spine in an active conformation and is preceded by a set of phosphorylated residues that are important for dimerization (Hu et al., 2013). In RET, W717 is preceded by a short sequence that engages the αC hydrophobic pocket that contains also the translocation site found in oncogenic RET fusions. To test the function of W717, we generated W717A and W717F mutants in the context of RET JM-KD and found that, contrary to W717F, the W717A mutant had a profound detrimental effect on RET phospho-tyrosine activity compared with WT (Figure 6A). These data indicate that W717 is required for RET catalytic activity. One plausible scenario is that W717 would be required for the proper alignment of the JM-proximal hydrophobic motif (DALKIL) to the αC hydrophobic patch (i.e., PIF-like pocket). Although correct in principle, this is unlikely based on the lack of effect in RET activity seen by alanine mutants targeting the DxLxI motif sequence (Figure S6), which suggest that residues farther up in the JM segment (e.g., Y687) make important contacts with the catalytic core (Figure 3). Alternatively, W717 would be required for docking onto and proper alignment of the R-spine in the active conformation, following the BRAF paradigm (Figure 6A). We hypothesize that mutating W717 by alanine and not by phenylalanine will perturb the R-spine side chain stacking and, as a consequence, impact on RET activity. Examination of the crystal structure (Figure 6B) revealed that in RET the R-spine is composed of four hydrophobic residues originating from the αF helix connecting N- and C-lobes; these residues include H872 (from the catalytic HRD motif), F893 (from the DFG motif), L779 (αC helix), and L790 (β-4 strand). W717 caps from the top the R-spine in a linear tetrad compatible with an active DFG in conformation of the kinase (Taylor and Kornev, 2011). It is further preceded by D714 adjacent to the fusion site between L712 and E713, which forms an important salt bridge with αC K780, a specific feature lacking in previously solved RET catalytic domain crystal structures. The combined effect of both W717 docking and the D714-K780 tether locks the hinge between the proximal JM segment and N-terminal residues of the catalytic core. In this scenario it is plausible also to hypothesize that perturbation of the hinge by the W717A mutant, contrary to hydrophobic motif DxLxIx alanine mutants, results in a non-compatible JM-proximal segment alignment with N-terminal residues of the catalytic core and a consequent alteration of the R-spine. How perturbation of the R-spine linear architecture results in catalytic inefficiency is likely an indirect effect on both the catalytic (C)-spine and the catalytically required K758 (β-3 strand)-E775 (αC helix)-D892 (DFG motif) tether, which links both spines and the nucleotide moiety (Figure 6C). From these data we conclude that W717 is an important residue for RET function by playing a role in the assembly of the JM hinge and R-spine.

Activation-Loop Serine Phosphorylation Is Required for RET Signaling In Vivo

S909 is a novel autoP site that arises from intrinsic dual-specificity kinase activity exhibited by RET in vitro (Figure 4D). Functional assays of a S909A mutant excluded a direct role of S909 on RET catalytic activity (Figures S4C and S4D). These results are consistent with data for mutants targeting RET KD tyrosine autoP sites (Figure 3B) and suggest phospho-S909 could act as a docking or alternatively a substrate site for effector proteins important for RET signaling. Given the high conservation of S909 in all RET sequences and its consistent occupancy, we assessed whether S909 had a role in RET downstream signaling. We therefore used a *Drosophila* Ret^{2B} (*dRet*^{2B}; *dRet M955T*) fly model to assess whether mutation at residue S946 (equivalent to human RET S909) could influence in vivo the aberrant phenotype promoted by oncogenic *dRet*^{2B}. We employed a pUAST-attB fly vector system to allow specific site insertion of the transgene into the fly genome (Bischof et al., 2007). Overexpression of *dRet*^{2B} in the developing eye using the glass multiple reporter (GMR) Gal4-815 promoter (GMR-Gal4-815 > *dRet*^{2B}) led to extensive mispatterning and positioning of ommatidia resulting in a "rough eye" phenotype in the adult fly compared with the driver-line control. When we generated a transgenic fly expressing a double mutant *dRet*$^{2B/S946A}$ (*dRet M955T/S946A*), the aberrant rough eye phenotype was completely rescued (Figure 7A). Further ectopic overexpression of *dRet*^{2B} in the peripodial cells of the developing wing epithelium under the control of the 765 promoter (*765>dRetM955T)* led to an increase in the number of aberrant veins (Figure 7B, arrows) within the adult wing (Figures 7B and 7C). As anticipated, ectopic expression of the double mutant in the developing wing (*ptc*765>*dRet M955T/S946A*) resulted in the abolition of the aberrant phenotype. To assess whether a signaling defect was associated with S946A (S909A in humans), we expressed ectopically dRET WT, M955T, and M955T/S946A in S2 insect cells, together with an *Actin* promoter-driven *Gal4* construct, and performed WB analyses. We observed a significant detrimental effect on the double mutant dRet M955T/S946A compared with oncogenic dRet M955T in downstream signaling as indicated by total phospho-tyrosine antibody, and also on dRet phosphorylation, as indicated by RET Y1015 and Y1062 phospho-specific antibodies (Figure 7D). These data indicate that an S909 phosphorylation event plays a crucial role in RET signaling in vivo. These results point

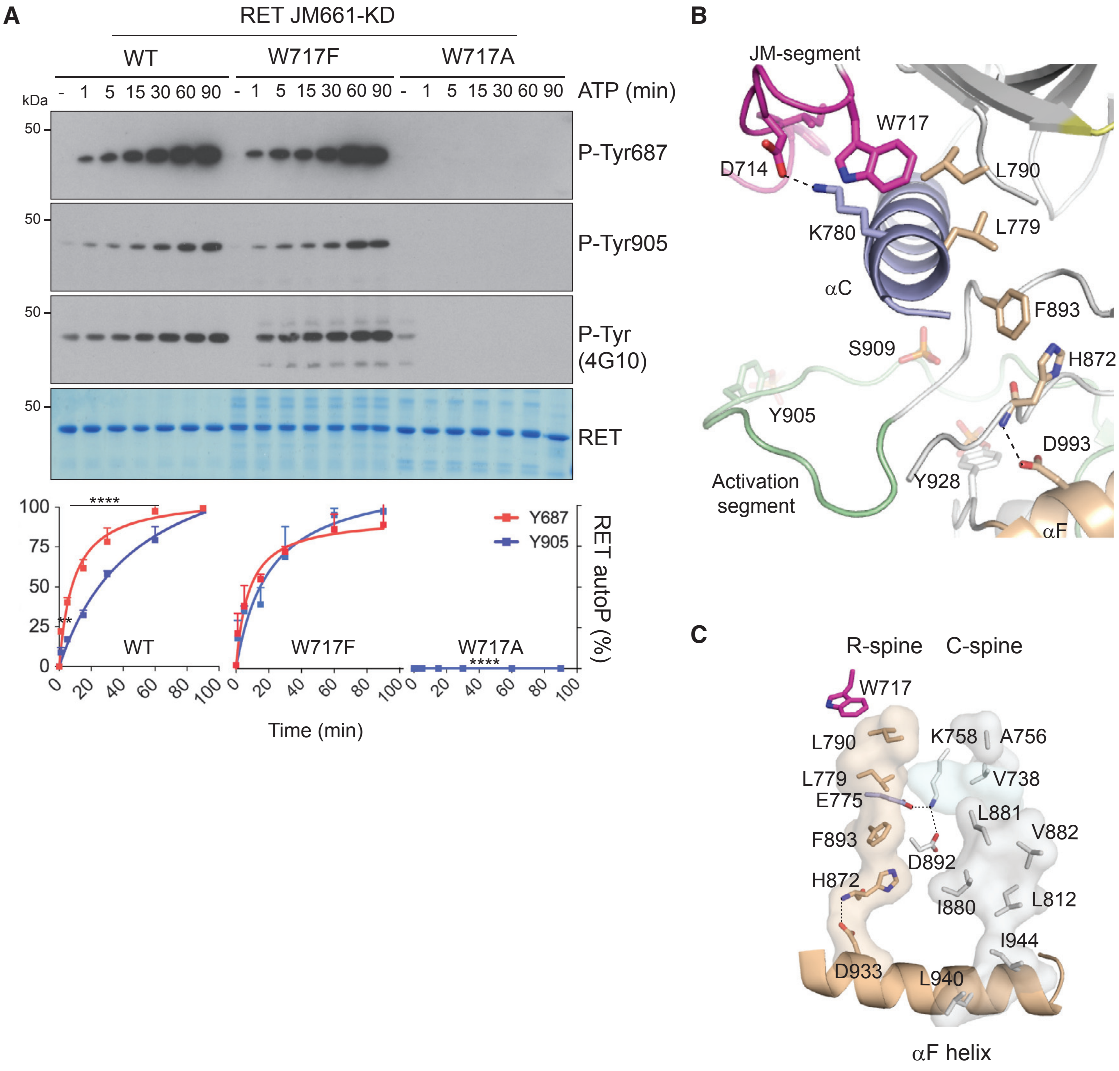

Figure 6. RET W717 Contributes to the Assembly of the JM Hinge and R-Spine

(A) WB analysis of in vitro time-course autoP assay using RET JM661-KD WT and indicated mutants (1 μM) after addition of ATP (5 mM) and $MgCl_2$ (10 mM) for 0–90 min using the indicated antibodies. Quantitation of RET phospho-Y687 and -Y905 signal is shown; data represent mean ± SEM, n = 3, ****p < 0.0001, **p = 0.0014, two-way ANOVA Bonferroni test.

(B) Cartoon representation of RET JM-KD crystal structure secondary structural elements, colored according to Figure 4A. Close-up of JM-hinge-composing residues (D714, K780, and W717) and R-spine-assembling residues (L779, L790, F893, H872, and D993) is shown; further selected residues and secondary structural elements are depicted as in Figure 4A. Some residues and structural elements have been omitted for clarity.

(C) View of the C- and R-spines of RET JM-KD crystal structure as per text (see Results and Discussion for R-spine); in addition, the catalytic triad K758 (β-3 strand)-E775 (αC helix)-D892 (DFG motif) in sticks representation and the nucleotide moiety represented by the PP1 inhibitor (soft blue surface) are shown. The larger C-spine of RET contains residues emanating from the hydrophobic αF helix, through to nucleotide and capping N-lobe residues (i.e., L940, I944, L812, I880, L881, V882, V738, and A756).

toward further complexity through interplay with RET phosphotyrosine sites, or alternatively as a docking and/or phospho-site for a yet unknown effector that impacts on RET signaling.

DISCUSSION

To identify unique features of RET tyrosine kinase activation, we have applied biochemical, structural, and biophysical analyses, together with an in vivo model for RET hyper-activation. We show that the JM segment functions to increase RET tyrosine KD activity without affecting substrate presentation in *trans*. Fully phosphorylated RET JM-KD appears rapidly, between 20 and 40 min, compared with the 80–120 min required for core RET KD (Figure 1B). This increased activity promoted by the JM segment, contrary to the EGFR (Jura et al., 2009; Red Brewer et al., 2009), does not appear to result from stable dimer formation in solution (Figure S1C) and is independent of “forced” dimerization through the presence of a GST tag (Figure 2D). Our results are

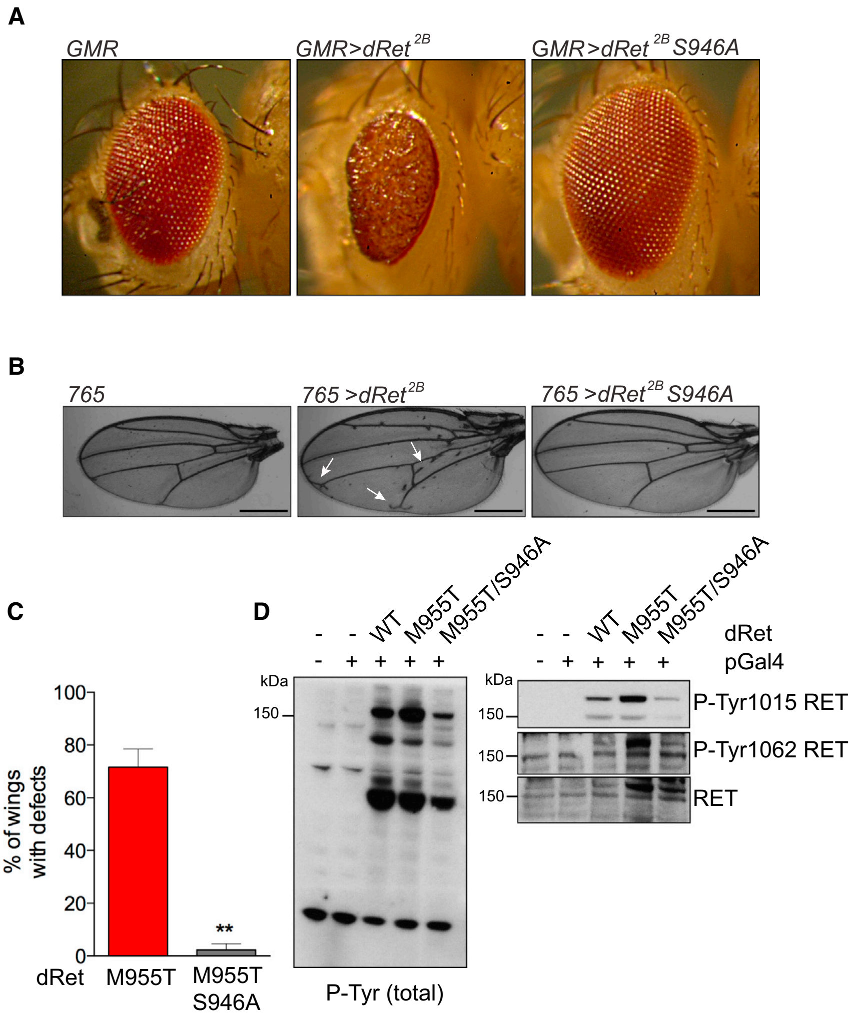

Figure 7. An Activation-Loop Serine Phospho-Site Is Required for RET Signaling In Vivo

(A) Ectopic expression of *dRet*^{2B} (*dRet M955T*) and *dRet*^{2B} *S946A* in the retina from the *GMR-Gal4 815* promoter.

(B) Expression of *dRet*^{2B} from the *Gal4 765* promoter led to ectopic vein formation. *S946A* mutation rescued the wing defects. Scale bar, 500 μM.

(C) Quantification of data shown in Figure 6B. The percentage of wings with ectopic veins was determined from three distinct transformants for each genotype. The number of individual flies counted from each transformant was 19, 30, and 23 for *dRet*^{2B} and 35, 17, and 23 for *dRet*^{2B} *S946A*. **$p < 0.01$, one-way ANOVA Bonferroni test.

(D) WB analyses of S2 insect cells ectopically expressing *dRet* WT, M955T, and M955T/S946A, together with an *Actin*-promoter-driven *Gal4* construct using the indicated antibodies.

consistent with VEGFR2, where in solution the kinetics of autoP is significantly enhanced by the JM region (Solowiej et al., 2009). Comparing these data with the slower overall phosphorylation kinetics of RET ICD (Plaza-Menacho et al., 2014a) suggests the C-terminal (CT) segment could act as a negative regulator of RET catalytic domain activity and restrain by competition JM segment activating input. The RET JM region appears not to play a *cis*-inhibitory role as observed for KIT and MET (Chan et al., 2003; Hubbard, 2004) but is likely to stabilize an active form of RET in a manner that is dependent on Y687. We argue that autoP is not prevented by a non-phosphorylated conformer of JM segment on Y687, but that timely phosphorylation of JM segment on Y687 leads to a conformation contributing to a more active RET kinase. This is supported by the observation that Y687 is required for the JM segment to rescue a catalytically deficient RET KD lacking both activation-loop tyrosines (Y900/905F). Furthermore, a RET JM-KD Y687E mutant (mimicking a constitutive phospho-Y687) showed a significant decrease on tyrosine kinase activity, which indicated that Y687 is a tightly regulated autoP site. We hypothesize that constitutive phosphorylation on Y687 results in a detrimental effect on activity because of the lack of required contacts between JM segment and RET catalytic core prior and during kinase activation (Figure S5B). Furthermore, the dependency seen by RET JM-KD, but not RET KD, on αC R770 implicates its side chain in engaging the JM segment to increase RET catalytic domain activity. Further evidence for the *cis* effect of the JM segment in RET activation includes: (1) phosphorylation rescue experiments in *trans* using as substrate catalytically deficient versions (i.e., K758M) of RET JM-KD and RET KD (Figure S3B) did not show significant differences because of the JM segment, and (2) the presence of the RET JM-segment effectively rescues an otherwise catalytically-deficient RET mutant bearing a double-tyrosine (Y900/905F) substitution in the activation-loop. Note this is contrary to the rescue experiment in *trans* of catalytically deficient K758M kinase versions, which cannot be rescued in *cis* by either JM segment (Figure S3) or oncogenic mutations (Plaza-Menacho et al., 2014a).

The trajectory of the proximal part of the RET JM-segment resembles to some extent that seen in the IR JM-KD crystal structure (PDB: 1P14) (Li et al., 2003). In the IR crystal structure, JM-Y984 docks into the αC hydrophobic patch in *cis* (adopting an equivalent position to RET F776) and forms a network of hydrogen bonds between residues from the αC helix and proximal JM segment. These interactions provide steric restraints preventing αC from assuming a catalytically competent position. A recent study has shown, however, that the JM-IR can also adopt a JM-out conformer contacting the αC of a second receptor molecule and is able to stabilize an active catalytic dimer (Cabail et al., 2015). This role for the IR JM-segment in *trans* tethers a quite distinctive symmetric active dimer compared with that observed for the asymmetric EGFR dimer (Jura et al., 2009). This recent JM-IR structure (PDB: 4XLV) shows how the JM-segment pivots about the equivalent residue to RET W717

(Hu et al., 2013) to make intermolecular contacts with the αC hydrophobic patch from a second molecule. This resembles in *trans* an extended stretch of the proximal JM region seen in our RET JM-KD crystal structure. Our interpretation is that in the absence of a stable RET JM-KD dimer in solution or in the crystal, the JM-segment collapses onto the αC helix in *cis* in the crystal lattice. Our data pointed also at the critical RET αC hydrophobic pocket as being sensitive to allosteric input from JM-segment elements, possibly including Y687. This hydrophobic pocket has the potential to be targeted by small molecules, because there are precedents for PDK1 where allosteric inhibitors against an equivalent site are already available (Busschots et al., 2012).

Chromosomal translocations involving the RET exons 12–21 are found in human thyroid and lung cancers (Nikiforov and Nikiforova, 2011; Plaza-Menacho et al., 2014b). These gene rearrangements fuse a variety of unrelated coiled-coil proteins within the same RET intron, thereby removing exons 1–11, including the JM segment. Our data are consistent with a scenario in which removing the JM segment, rather than eliminating an autoinhibitory element, replaces it with a more potent dimerizing motif that stabilizes a RET dimer independently of ligand and transmembrane region. For the IR, its JM segment extends away from the kinase core pivoting about a conserved VPDEWE motif to engage a second kinase molecule via contacts to the αC helix. A network of salt-bridge interactions at the pivot point involves the VPDEWE motif to help stabilize a conformation associated with an activated IR tyrosine kinase. The equivalent sequence for RET, EDPKWE, contains the fusion site (between L712 and E713) of many RET translocations that eliminate RET exons 1–11 (Nikiforov and Nikiforova, 2011). Such fusions add a dimeric coiled-coil region just prior to D714 and W717 that would lock permanently the JM hinge and R-spine into a DFG-in conformer resulting in a hyperactive RET, no longer localized at the plasma membrane. These findings have important drug discovery and therapeutic implications as perturbation of the JM hinge and consequent effect on adequate R-spine assembly could be a new drug-targetable strategy against oncogenic RET.

The JM-KD crystal structure shows two unexpected phosphorylation sites, Y928 and S909, both invariant RET residues. Both are in close proximity and engage basic residues otherwise found in the core RET KD structures engaged by phospho-Y905 (Figures 4A and 4B; Figure S4B). In particular, the unconventional activation-loop S909 phospho-site engages the HRD motif in what it seems to be a unique active conformation promoting both regulatory-spine assembly and accessibility to phosphotyrosine binding modules. As a consequence, Y905 is displaced, adopting a solvent-accessible conformer competent for a signaling function rather than playing an activating role.

Analyses of RET sequences flanking the invariant protein kinase RD motif establish it as tyrosine kinase (HRDLAARN or HRDLRAAN) rather than a serine-threonine kinase (H/YRDLXXN) (Hanks et al., 1988; Lindberg et al., 1992). There are precedents for dual-specificity kinase activity among the cyclin-dependent kinase (CDK), mitogen-activated protein kinase (MAPK), glycogen synthase kinase (GSK3), CDC-like kinase (CLK) group of protein kinases (CMGC) that include the mitogen-activated protein kinase (MAPK) and DYRK kinase members. The latter examples phosphorylate exclusively serine and threonine side chains in their substrates but are able to autoP on tyrosine (Himpel et al., 2000). More recently, a non-receptor tyrosine kinase Syk has been shown to exhibit dual-specificity kinase activity (Heizmann et al., 2010). In our study, robust biochemical examination revealed that S909 is an autoP site that arises from an intrinsic RET dual-specificity kinase activity. This is consistent with a recent study reporting that RET can phosphorylate AP2 in *trans* on threonine (Bagheri-Yarmand et al., 2015). This dual-specific activity can generate phospho-S909 in vitro. However, phosphorylation on RET S909 was not catalytically required (Figures S4C–S4E), consistent with other serine-to-alanine mutants targeting the JM segment (Figure S5C). Further efforts are needed to establish whether phospho-S909 can act as a docking for a yet-unknown effector protein involved in RET signaling. Interestingly, when we evaluated an RET S909D mutant, a marked increase in RET JM-KD phospho-tyrosine activity was observed (Figure S5B). These data suggest that a phosphorylation event on S909 in *trans* can contribute to RET tyrosine kinase activity and signaling in vivo. To explore further this hypothesis, we employed an in vivo model and found that mutating this residue in *Drosophila* Ret (*dRet*) has a deleterious effect on the *dRet^{2B}* (*dRet M955T*) oncogenic phenotype, a well-established model of transformation. In particular, *dRet M955T/S946A* efficiently rescued the phenotype of *dRet^{2B}* using alternative promoters.

Overall, our structure-function analyses and in vivo experiments have revealed complex elements in the mechanism of RET activation and signaling. Allosteric inputs from the JM-segment and activation-loop S909 contribute to kinase function. We show that phospho-S909 is an autoP site arising from an intrinsic dual-specificity RET kinase activity and appears to play key roles in oncogenic signaling. Our study also suggests that targeting the αC hydrophobic pocket together with the JM hinge using small molecules to manipulate RET kinase activity may be a productive approach for either blocking oncogenic forms of RET or stimulating RET activity in Hirschsprung's disease (HSCR) and neurodegenerative Parkinson's disease (PD).

EXPERIMENTAL PROCEDURES

Expression and Purification of Recombinant Protein

Protein expression was carried out using Sf9 insect cells following a previously described protocol (Knowles et al., 2006). Codon optimized human RET9 isoform intracellular domain (ICD residues 661–1072), different lengths versions of the RET JM-KD (661 to 698–1012) and RET KD core (705–1013) WT, and the indicated mutants proteins were purified following a protocol previously described (Plaza-Menacho et al., 2014a).

Mass Spectrometric Label-free Quantitation

Mass spectrometry procedures were performed as previously described (Plaza-Menacho et al., 2014a).

Autophosphorylation Assays, SDS-PAGE, and Western Blotting

Unless otherwise indicated, time-course autoP assays were performed with recombinant purified protein as previously described (Plaza-Menacho et al., 2014a). Western blotting was performed with the indicated antibodies as previously described (Plaza-Menacho et al., 2010, 2011). A specific antibody against RET phospho-S909 epitope (pSQG) was from Cell Signaling. Data represent at least two to six independent experiments (n) using different protein preparations. In addition to the quantitation of WB data shown on Figures 4D and 6A, further quantitation of the indicated WB analyses can also be found in the Supplemental Information.

Enzymatic Kinase Assays

Enzyme kinetic experiments were performed as previously described (Plaza-Menacho et al., 2014a).

ITC

ITC experiments were performed as previously described (Plaza-Menacho et al., 2014a).

Dynamic Light Scattering and ThermoFluor Assays

To determine protein stability, we performed thermal shifts assays as previously described (Plaza-Menacho et al., 2010, 2014a). Molecular weight determination in solution was performed by DLS using different RET protein concentrations.

Crystallization, Diffraction, Data Collection, and Processing

Crystals of the phosphorylated RET JM-catalytic domain (residues 659–1013) were grown at 22°C by vapor diffusion in sitting drops containing crystal 1 (5FM2), 1 μL protein stock solution (6 mg/ml) mixed with 1 μL reservoir solution (1.5 M ammonium sulfate, 0.1 M BIS-TRIS propane [pH 7.0]); the protein stock solution also contained 2.5 mM ATP and 5 mM $MgCl_2$. Crystal 2 (5FM3) comprised 0.8 μL protein stock solution (5 mg/ml) mixed with 0.8 μL reservoir solution (1.2 ammonium sulfate, 0.1 M tri-sodium citrate [pH 5.43]). The crystals were cryoprotected in 25% glycerol in reservoir solution for several minutes and flash frozen in liquid nitrogen, and X-ray datasets were collected at the I-24 beamline of the Diamond Light Source Synchrotron (Oxford, UK). Data collection and refinement statistics are summarized in Table 1. The dataset was indexed with MOSFLM and scaled with SCALA (Winn et al., 2011). Molecular replacement was carried out using the atomic coordinates of the phosphorylated RET KD (PDB: 2IVT) in PHASER (McCoy et al., 2007). Refinement was carried out by using Phenix (Adams et al., 2010). Model building was carried out in COOT (Emsley et al., 2010). Model validation used PROCHECK (Vaguine et al., 1999), and figures were prepared using the graphics program PYMOL (http://www.pymol.org).

Drosophila Experiments

pUASTattB-*dRetM955T* (*dRet*^{2B}) and double mutant pUASTattB-*dRetM955T/S946A* constructs were generated by site-directed mutagenesis using the following primers: M955T forward 5′-GTGCCCGTCAAGTGGACG GCTCCGGA-3′, M955T reverse 5′- TCCGGAGCCGTCCACTTGACGGGCAC-3′, S946A forward 5′- GCCTATTTAAAGAGAGCCCGAGATCGTGTGCCC-3′, and S946A reverse 5′- GGGCACACGATCTCGGGCTCTCTTTAAATAGGC. Transgenic flies were generated using P-element-mediated (pUAST) transgenesis by BestGene. *Drosophila* stocks and crosses were maintained at 25°C, unless stated otherwise. For ectopic expression of the various transgenes in the developing *Drosophila* wing, *dRet* transgenic flies were crossed with the Gal4 C-765 driver (36523, Bloomington). Adult wings were dissected, mounted, and imaged at 4× magnification using the EVOS cell imaging system. For ectopic expression of the *dRet*^{2B} and *dRet*^{2B} *S946A* in the developing eye, the transgenic flies were crossed with the GMR-Gal4 815 (weak) driver and maintained at 18°C. Eye phenotypes were analyzed by light microscopy of whole mounts.

Ectopic Expression in S2 Cells

pUASTattB-*dRet* WT, M955T, and M955T/S947A constructs (400 ng) were co-transfected together with an *Actin*-promoter-driven *Gal4* plasmid (400 ng) as indicated into S2 cells using Effectene and following manufacturer's instructions. Data represent three independent experiments.

Statistical Analyses

Graphs and statistical analyses were done using Prism GraphPad.

ACCESSION NUMBERS

The crystallographic coordinates and structure factors for the RET JM-KD crystal structures reported in this paper are PDB: 5FM3 and 5FM2, respectively (http://www.pdb.org).

SUPPLEMENTAL INFORMATION

Supplemental Information includes six figures and can be found with this article online at http://dx.doi.org/10.1016/j.celrep.2016.11.061.

AUTHOR CONTRIBUTIONS

I.P.-M. and N.Q.M. planned the project, designed experiments, analyzed the data, and wrote the paper. I.P.-M. performed all biochemical and crystallographic experiments, assembled the initial draft of the paper, and prepared all figures. K.B. performed the mass spectrometry analyses. A.B. and R.C. assisted directly with baculovirus production. R.B. and M.O. performed *Drosophila* experiments under supervision of I.P.-M. and P.M. S.M. assisted with data processing and structure determination. R.J.M.-T. performed the ITC experiments.

ACKNOWLEDGMENTS

We thank our Lincoln's Inn Fields Laboratories (Francis Crick Institute) colleagues Sara Kisakye Nambozo, Roger George, and Svend Kjaer (Protein Production Facility) for their technical assistance in the production of baculoviruses; Nicola O'Reilly (Peptide Production Facility) for timely supply of peptides; and Andrew Purkiss-Trew (Structural Biology Laboratory) for helping with data collection and structure determination. We also thank Tilman Schirmer (Biozentrum, University of Basel) for helpful comments on the manuscript and Ross Cagan (Icahn School of Medicine, Mount Sinai, NY) for providing initial pUAST-*dRet* plasmids. We thank Diamond Light Source Synchrotron (Oxford, UK) for allowing X-ray dataset collection at the I-24 beamline. N.Q.M. acknowledges that this work was supported by the Francis Crick Institute, which receives its core funding from Cancer Research UK (FC001115), the UK Medical Research Council (FC001115) and the Wellcome Trust (FC001115); by the NCI/NIH (grant reference 5R01CA197178); by the Association for Multiple Endocrine Neoplasia Disorders MTC Research Fund. P.M. acknowledges NHS funding to the NIHR Biomedical Research Centre.

Received: March 22, 2016
Revised: October 2, 2016
Accepted: November 20, 2016
Published: December 20, 2016

REFERENCES

Adams, P.D., Afonine, P.V., Bunkóczi, G., Chen, V.B., Davis, I.W., Echols, N., Headd, J.J., Hung, L.W., Kapral, G.J., Grosse-Kunstleve, R.W., et al. (2010). PHENIX: a comprehensive Python-based system for macromolecular structure solution. Acta Crystallogr. D Biol. Crystallogr. *66*, 213–221.

Airaksinen, M.S., Titievsky, A., and Saarma, M. (1999). GDNF family neurotrophic factor signaling: four masters, one servant? Mol. Cell. Neurosci. *13*, 313–325.

Asai, N., Fukuda, T., Wu, Z., Enomoto, A., Pachnis, V., Takahashi, M., and Costantini, F. (2006). Targeted mutation of serine 697 in the Ret tyrosine kinase causes migration defect of enteric neural crest cells. Development *133*, 4507–4516.

Bae, J.H., and Schlessinger, J. (2010). Asymmetric tyrosine kinase arrangements in activation or autophosphorylation of receptor tyrosine kinases. Mol. Cells *29*, 443–448.

Bagheri-Yarmand, R., Sinha, K.M., Gururaj, A.E., Ahmed, Z., Rizvi, Y.Q., Huang, S.C., Ladbury, J.E., Bogler, O., Williams, M.D., Cote, G.J., and Gagel, R.F. (2015). A novel dual kinase function of the RET proto-oncogene negatively regulates activating transcription factor 4-mediated apoptosis. J. Biol. Chem. *290*, 11749–11761.

Biondi, R.M., Cheung, P.C., Casamayor, A., Deak, M., Currie, R.A., and Alessi, D.R. (2000). Identification of a pocket in the PDK1 kinase domain that interacts with PIF and the C-terminal residues of PKA. EMBO J. *19*, 979–988.

Bischof, J., Maeda, R.K., Hediger, M., Karch, F., and Basler, K. (2007). An optimized transgenesis system for Drosophila using germ-line-specific phiC31 integrases. Proc. Natl. Acad. Sci. USA *104*, 3312–3317.

Busschots, K., Lopez-Garcia, L.A., Lammi, C., Stroba, A., Zeuzem, S., Piiper, A., Alzari, P.M., Neimanis, S., Arencibia, J.M., Engel, M., et al. (2012). Substrate-selective inhibition of protein kinase PDK1 by small compounds that bind to the PIF-pocket allosteric docking site. Chem. Biol. *19*, 1152–1163.

Cabail, M.Z., Li, S., Lemmon, E., Bowen, M.E., Hubbard, S.R., and Miller, W.T. (2015). The insulin and IGF1 receptor kinase domains are functional dimers in the activated state. Nat. Commun. *6*, 6406.

Chan, P.M., Ilangumaran, S., La Rose, J., Chakrabartty, A., and Rottapel, R. (2003). Autoinhibition of the kit receptor tyrosine kinase by the cytosolic juxtamembrane region. Mol. Cell. Biol. *23*, 3067–3078.

Chen, H., Ma, J., Li, W., Eliseenkova, A.V., Xu, C., Neubert, T.A., Miller, W.T., and Mohammadi, M. (2007). A molecular brake in the kinase hinge region regulates the activity of receptor tyrosine kinases. Mol. Cell *27*, 717–730.

Emsley, P., Lohkamp, B., Scott, W.G., and Cowtan, K. (2010). Features and development of Coot. Acta Crystallogr. D Biol. Crystallogr. *66*, 486–501.

Fukuda, T., Kiuchi, K., and Takahashi, M. (2002). Novel mechanism of regulation of Rac activity and lamellipodia formation by RET tyrosine kinase. J. Biol. Chem. *277*, 19114–19121.

Hanks, S.K., Quinn, A.M., and Hunter, T. (1988). The protein kinase family: conserved features and deduced phylogeny of the catalytic domains. Science *241*, 42–52.

Heizmann, B., Reth, M., and Infantino, S. (2010). Syk is a dual-specificity kinase that self-regulates the signal output from the B-cell antigen receptor. Proc. Natl. Acad. Sci. USA *107*, 18563–18568.

Himpel, S., Tegge, W., Frank, R., Leder, S., Joost, H.G., and Becker, W. (2000). Specificity determinants of substrate recognition by the protein kinase DYRK1A. J. Biol. Chem. *275*, 2431–2438.

Hu, J., Stites, E.C., Yu, H., Germino, E.A., Meharena, H.S., Stork, P.J., Kornev, A.P., Taylor, S.S., and Shaw, A.S. (2013). Allosteric activation of functionally asymmetric RAF kinase dimers. Cell *154*, 1036–1046.

Hubbard, S.R. (1997). Crystal structure of the activated insulin receptor tyrosine kinase in complex with peptide substrate and ATP analog. EMBO J. *16*, 5572–5581.

Hubbard, S.R. (2004). Juxtamembrane autoinhibition in receptor tyrosine kinases. Nat. Rev. Mol. Cell Biol. *5*, 464–471.

Jura, N., Endres, N.F., Engel, K., Deindl, S., Das, R., Lamers, M.H., Wemmer, D.E., Zhang, X., and Kuriyan, J. (2009). Mechanism for activation of the EGF receptor catalytic domain by the juxtamembrane segment. Cell *137*, 1293–1307.

Jura, N., Zhang, X., Endres, N.F., Seeliger, M.A., Schindler, T., and Kuriyan, J. (2011). Catalytic control in the EGF receptor and its connection to general kinase regulatory mechanisms. Mol. Cell *42*, 9–22.

Kannan, N., Haste, N., Taylor, S.S., and Neuwald, A.F. (2007). The hallmark of AGC kinase functional divergence is its C-terminal tail, a cis-acting regulatory module. Proc. Natl. Acad. Sci. USA *104*, 1272–1277.

Knowles, P.P., Murray-Rust, J., Kjaer, S., Scott, R.P., Hanrahan, S., Santoro, M., Ibáñez, C.F., and McDonald, N.Q. (2006). Structure and chemical inhibition of the RET tyrosine kinase domain. J. Biol. Chem. *281*, 33577–33587.

Kovacs, E., Zorn, J.A., Huang, Y., Barros, T., and Kuriyan, J. (2015). A structural perspective on the regulation of the epidermal growth factor receptor. Annu. Rev. Biochem. *84*, 739–764.

Lemmon, M.A., and Schlessinger, J. (2010). Cell signaling by receptor tyrosine kinases. Cell *141*, 1117–1134.

Lemmon, M.A., Schlessinger, J., and Ferguson, K.M. (2014). The EGFR family: not so prototypical receptor tyrosine kinases. Cold Spring Harb. Perspect. Biol. *6*, a020768.

Li, S., Covino, N.D., Stein, E.G., Till, J.H., and Hubbard, S.R. (2003). Structural and biochemical evidence for an autoinhibitory role for tyrosine 984 in the juxtamembrane region of the insulin receptor. J. Biol. Chem. *278*, 26007–26014.

Lindberg, R.A., Quinn, A.M., and Hunter, T. (1992). Dual-specificity protein kinases: will any hydroxyl do? Trends Biochem. Sci. *17*, 114–119.

Lougheed, J.C., Chen, R.H., Mak, P., and Stout, T.J. (2004). Crystal structures of the phosphorylated and unphosphorylated kinase domains of the Cdc42-associated tyrosine kinase ACK1. J. Biol. Chem. *279*, 44039–44045.

McCoy, A.J., Grosse-Kunstleve, R.W., Adams, P.D., Winn, M.D., Storoni, L.C., and Read, R.J. (2007). Phaser crystallographic software. J. Appl. Cryst. *40*, 658–674.

Nikiforov, Y.E., and Nikiforova, M.N. (2011). Molecular genetics and diagnosis of thyroid cancer. Nat. Rev. Endocrinol. *7*, 569–580.

Perrinjaquet, M., Vilar, M., and Ibáñez, C.F. (2010). Protein-tyrosine phosphatase SHP2 contributes to GDNF neurotrophic activity through direct binding to phospho-Tyr687 in the RET receptor tyrosine kinase. J. Biol. Chem. *285*, 31867–31875.

Plaza-Menacho, I., Burzynski, G.M., de Groot, J.W., Eggen, B.J., and Hofstra, R.M. (2006). Current concepts in RET-related genetics, signaling and therapeutics. Trends Genet. *22*, 627–636.

Plaza-Menacho, I., Morandi, A., Robertson, D., Pancholi, S., Drury, S., Dowsett, M., Martin, L.A., and Isacke, C.M. (2010). Targeting the receptor tyrosine kinase RET sensitizes breast cancer cells to tamoxifen treatment and reveals a role for RET in endocrine resistance. Oncogene *29*, 4648–4657.

Plaza-Menacho, I., Morandi, A., Mologni, L., Boender, P., Gambacorti-Passerini, C., Magee, A.I., Hofstra, R.M., Knowles, P., McDonald, N.Q., and Isacke, C.M. (2011). Focal adhesion kinase (FAK) binds RET kinase via its FERM domain, priming a direct and reciprocal RET-FAK transactivation mechanism. J. Biol. Chem. *286*, 17292–17302.

Plaza-Menacho, I., Barnouin, K., Goodman, K., Martínez-Torres, R.J., Borg, A., Murray-Rust, J., Mouilleron, S., Knowles, P., and McDonald, N.Q. (2014a). Oncogenic RET kinase domain mutations perturb the autophosphorylation trajectory by enhancing substrate presentation in trans. Mol. Cell *53*, 738–751.

Plaza-Menacho, I., Mologni, L., and McDonald, N.Q. (2014b). Mechanisms of RET signaling in cancer: current and future implications for targeted therapy. Cell. Signal. *26*, 1743–1752.

Red Brewer, M., Choi, S.H., Alvarado, D., Moravcevic, K., Pozzi, A., Lemmon, M.A., and Carpenter, G. (2009). The juxtamembrane region of the EGF receptor functions as an activation domain. Mol. Cell *34*, 641–651.

Solowiej, J., Bergqvist, S., McTigue, M.A., Marrone, T., Quenzer, T., Cobbs, M., Ryan, K., Kania, R.S., Diehl, W., and Murray, B.W. (2009). Characterizing the effects of the juxtamembrane domain on vascular endothelial growth factor receptor-2 enzymatic activity, autophosphorylation, and inhibition by axitinib. Biochemistry *48*, 7019–7031.

Takahashi, M., Asai, N., Iwashita, T., Isomura, T., Miyazaki, K., and Matsuyama, M. (1993). Characterization of the ret proto-oncogene products expressed in mouse L cells. Oncogene *8*, 2925–2929.

Taylor, S.S., and Kornev, A.P. (2011). Protein kinases: evolution of dynamic regulatory proteins. Trends Biochem. Sci. *36*, 65–77.

Thiel, K.W., and Carpenter, G. (2007). Epidermal growth factor receptor juxtamembrane region regulates allosteric tyrosine kinase activation. Proc. Natl. Acad. Sci. USA *104*, 19238–19243.

Thompson, E.E., Kornev, A.P., Kannan, N., Kim, C., Ten Eyck, L.F., and Taylor, S.S. (2009). Comparative surface geometry of the protein kinase family. Protein Sci. *18*, 2016–2026.

Vaguine, A.A., Richelle, J., and Wodak, S.J. (1999). SFCHECK: a unified set of procedures for evaluating the quality of macromolecular structure-factor data and their agreement with the atomic model. Acta Crystallogr. D Biol. Crystallogr. *55*, 191–205.

Winn, M.D., Ballard, C.C., Cowtan, K.D., Dodson, E.J., Emsley, P., Evans, P.R., Keegan, R.M., Krissinel, E.B., Leslie, A.G., McCoy, A., et al. (2011). Overview of the CCP4 suite and current developments. Acta Crystallogr. D Biol. Crystallogr. *67*, 235–242.

Zhang, X., Gureasko, J., Shen, K., Cole, P.A., and Kuriyan, J. (2006). An allosteric mechanism for activation of the kinase domain of epidermal growth factor receptor. Cell *125*, 1137–1149.

Cell Reports, Volume *17*

Supplemental Information

RET Functions as a Dual-Specificity Kinase that Requires Allosteric Inputs from Juxtamembrane Elements

Iván Plaza-Menacho, Karin Barnouin, Rachael Barry, Annabel Borg, Mariam Orme, Rakhee Chauhan, Stephane Mouilleron, Rubén J. Martínez-Torres, Pascal Meier, and Neil Q. McDonald

Figure S1 Plaza-Menacho et al.

A B

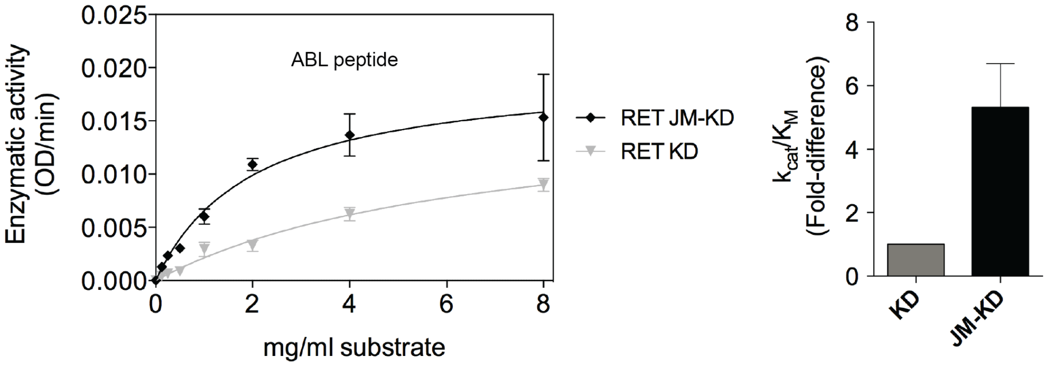

C

	Mw (kDa)	T_m (°C)	K_d (μM)	k_{cat} (min^{-1})	K_M (μM)	k_{cat}/K_M (min^{-1} μM^{-1})
**JM-KD**	41.6	41.0	37.5 ± 3	8.84 ± 0.03	305.0 ± 54	0.029
**KD**	33.9	42.2	64.3 ± 10	1.56 ± 0.15	269.6 ± 55	0.0058

D

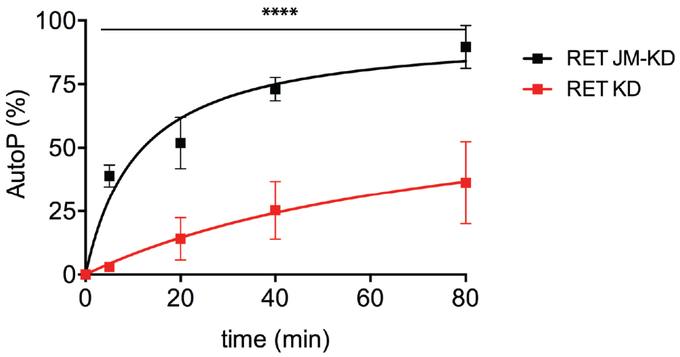

Figure S1 (related to Figure 1)

The juxtamembrane (JM) segment enhances RET catalytic domain activity.

(A-B) Enzymatic activity of RET JM-KD and RET KD measured against increasing concentrations of an exogenous ABL derived peptide substrate (sequence EAIYAAPFAKKK) showed increased catalytic efficiency (larger k_{cat}/K_M constant) towards the peptide-substrate. These data indicate increased RET catalytic activity promoted by the JM-region. Data represent mean ± standard error of the mean (SEM) from 2 experiments with 2-3 replicates each.

(C) Experimentally determined molecular weight (Mw), melting temperatures (T_m), dissociation constants (K_d) and enzyme kinetics (k_{cat}, K_M, k_{cat}/K_M for ATP) obtained by dynamic light scattering (DLS), ThermoFluor, ITC and enzymatic assays, respectively, as described in experimental procedures.

(D) Quantitation of WB data of figure 1B. Data represent the mean of phospho-tyrosine antibody (4G10) signal (i.e. autophosphorylation, percentage) normalized to total protein ± SEM, n=6. Statistics for RET JM-KD versus KD, **** $p<0.0001$ two-way Anova Bonferroni test.

Figure S2 Plaza-Menacho et al.

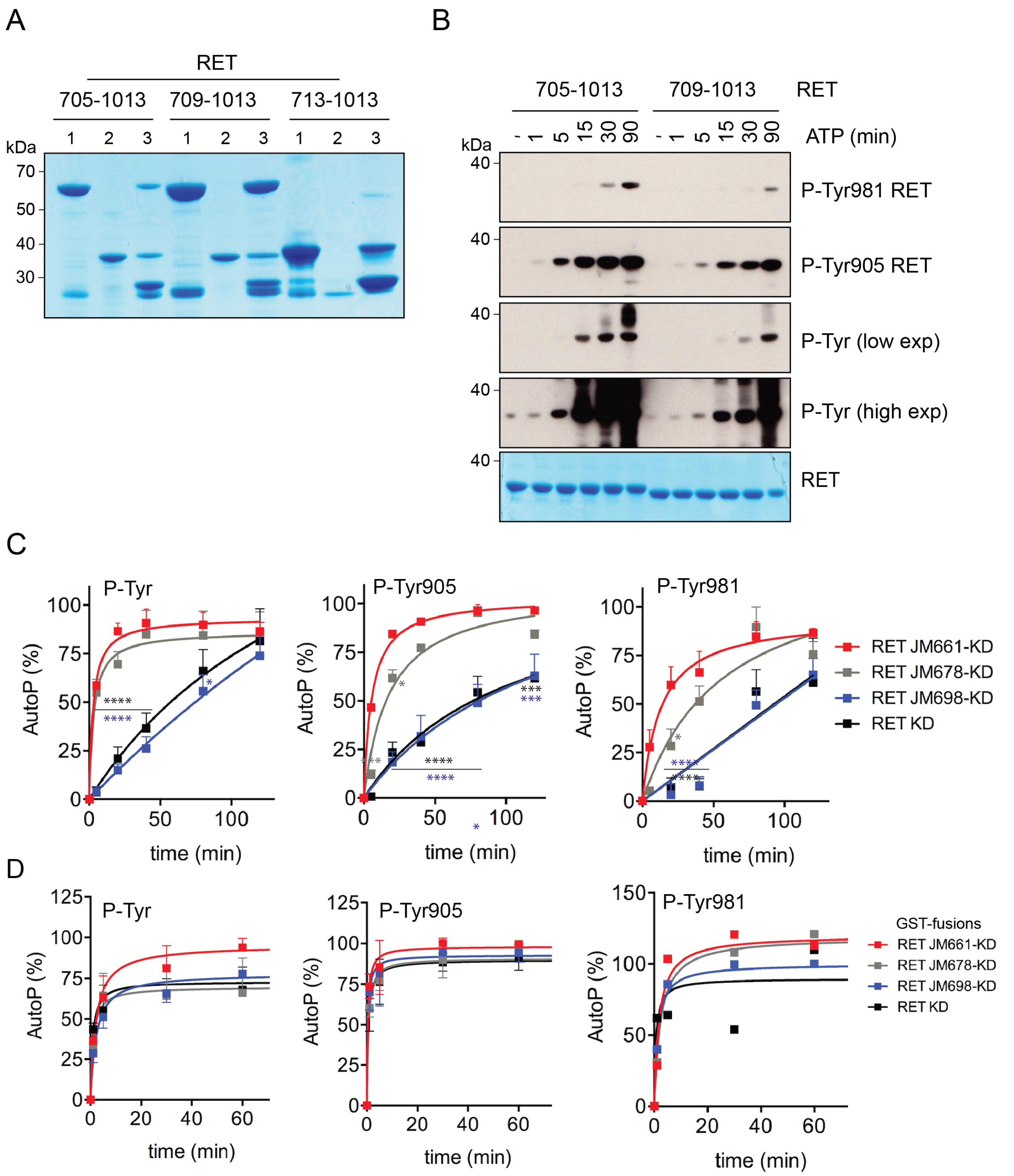

Figure S2 (related to figure 2)

Impact of fine deletions proximal to JM-segment and core kinase domain boundary and quantitation of figure 2 autophosphorylation assays.

Deletions targeting the transition from the JM-segment to the beginning of the RET catalytic core from residues 705 to 712 were evaluated in expression analysis and in phosphorylation assays.

(A) While recombinant RET catalytic domain starting at residue 709 was stable in solution, the construct starting from residue 713 gave rise to an unstable protein and

did not express properly. Lane 1 indicates GST-fusion protein in beads, lane 2 eluted protein after protease cleavage and lane 3 beads after cleavage.

(B) In phosphorylation assays the truncated RET starting at residue 709 displayed slower kinetics of autophosphorylation compared to RET KD starting at residue 705, as indicated by the different antibodies.

(C) Quantitation of WB data of figure 2C. Data represent the mean of autophosphorylation (percentage) ± SEM of the indicated antibodies normalized to total protein, n=3. Statistics * $p< 0.05$, *** $p<0.001$, **** $p<0.0001$ two- way Anova Bonferroni test versus control (WT).

(D) Quantitation of WB data of figure 2D. Data represent the mean of autophosphorylation (percentage) ± SEM of the indicated antibodies normalized to total protein, n=2.

Figure S3 Plaza-Menacho et al.

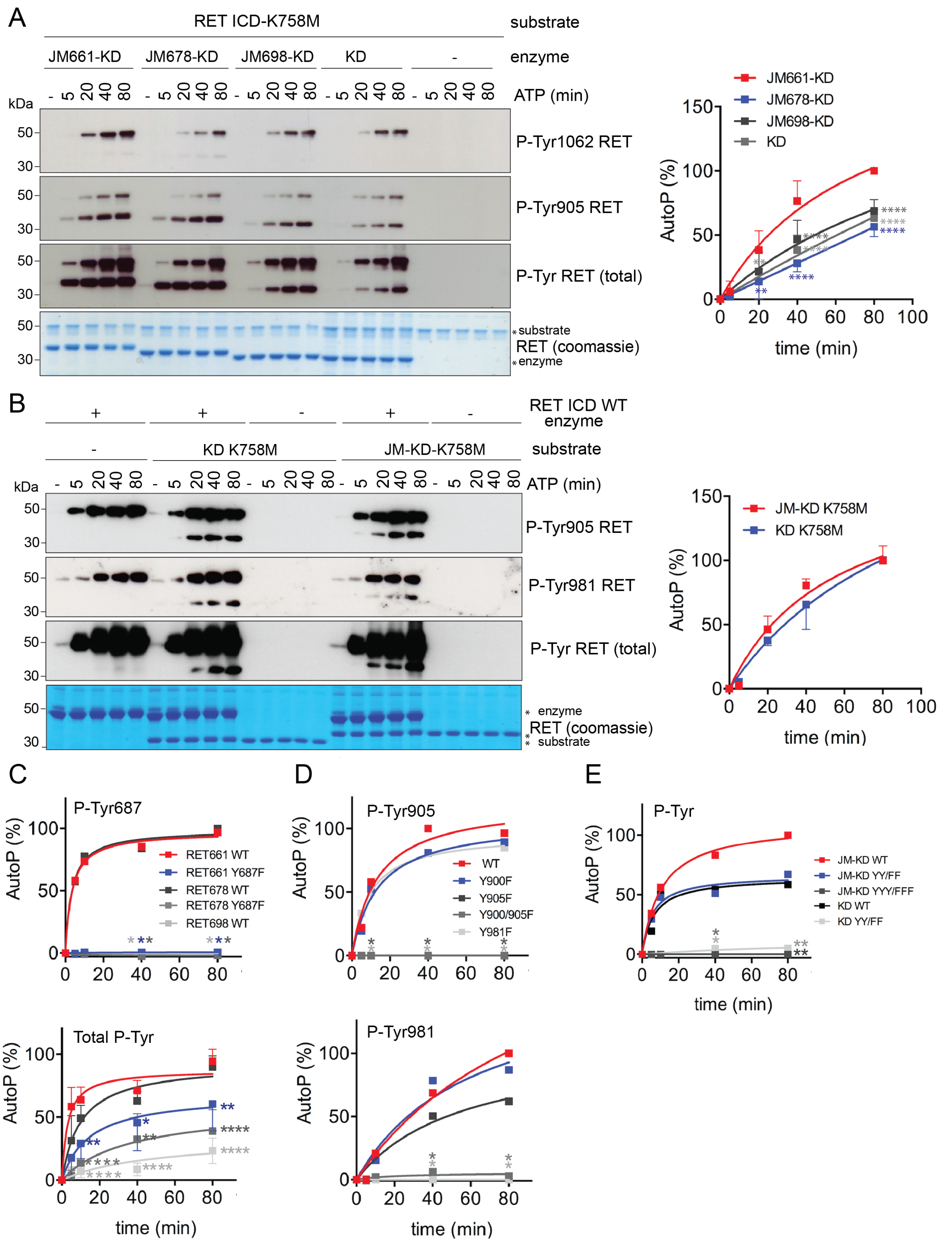

Figure S3 (related to figure 2 and 3)

Autophosphorylation rescue experiments in trans by RET JM-KD and RET KD and quantitation of RET autophosphorylation assays figure 3.

(A) To assess whether the JM-segment enhances the enzymatic properties of RET kinase, phosphorylation rescue experiments in trans using a catalytically impaired RET intracellular domain (ICD K758M) as a substrate were performed. Comparison between active RET JM-KD with different lengths of the JM-segment and RET KD core demonstrated an activating role for the JM terminal amino acid segment comprising residues 661-677. WB data quantitation of figure S3A, right panel. Data represent the mean of total phospho-tyrosine signal (i.e. autophosphorylation, percentage) normalized to total protein ± SEM, n=2. Statistics **** $p<0.0001$, ** $p<0.01$ two- way Anova Bonferroni test versus control (JM661-KD).

(B) To investigate whether the JM-region impacts on the substrate presentation properties of the receptor the reciprocal experiment was performed. Catalytically impaired RET K758M both JM-KD and KD core were used as substrates. Phospho-rescue experiments in trans by active RET ICD revealed that the JM-region does not affect the substrate properties of RET kinase, as no significant differences were observed between the two substrate variants. Note that enzyme variants used in upper panel (see lower bands) recapitulate data from in situ autophosphorylation assays (see figures 1 and 2). Quantitation of WB data of figure S3B, right panel. Data represent the mean of total phospho-tyrosine signal (i.e. autophosphorylation, percentage) normalized to total protein ± SEM, n=2.

(C) Quantitation of WB data of figure 3A. Data represent the mean of phospho-Tyr687 (upper panel, n=1) and total phospho-tyrosine signal (lower panel n=3) percentage normalized to total protein ± SEM. Statistics **** $p<0.0001$, ** $p<0.01$, * $p<0.5$, two- way Anova Bonferroni test versus control (JM661-KD).

(D) Quantitation of WB data of figure 3B. Data represent the mean of phospho-Tyr905 (upper panel) and phospho-Tyr981 (lower panel) normalized to total protein (n=1). Statistics **** $p<0.0001$, ** $p<0.01$, * $p<0.5$, two- way Anova Bonferroni test versus control (JM661-KD).

(E) Quantitation of WB data of figure 3C. Data represent the mean of total phospho-tyrosine antibody (4G10) signal (n=1) normalized to total protein. Statistics ** $p<0.01$, * $p<0.5$, two- way Anova Bonferroni test versus control (JM661-KD).

Figure S4 Plaza-Menacho et al.

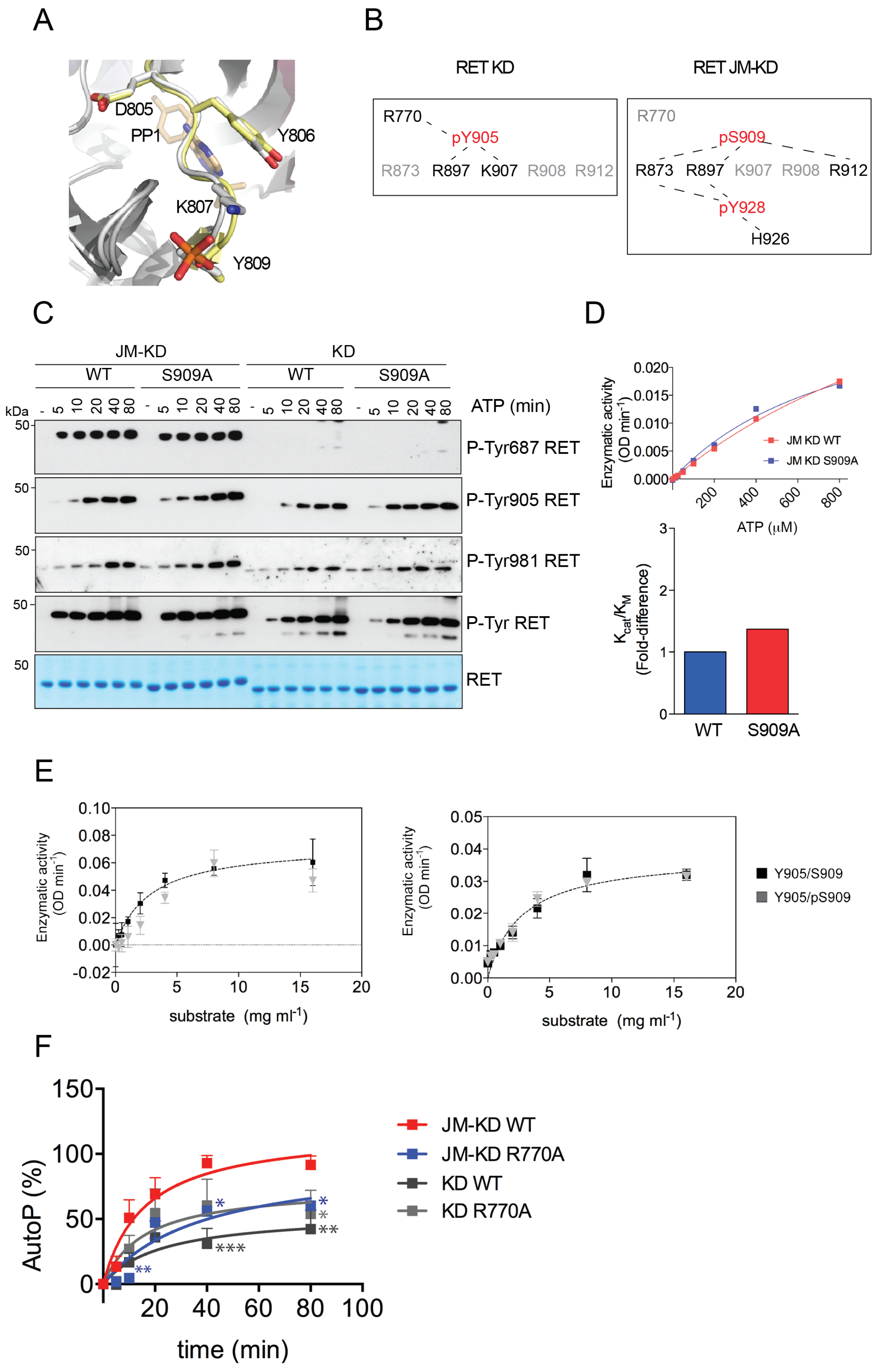

Figure S4 (related to figure 4)

Structural details of RET JM-KD crystal structure, functional evaluation of S909 on RET catalytic domain activity and quantitation of RET autophosphorylation assay figure 4E.

(A) Detailed view of the hinge region of RET JM-KD (PDB ID code 5FM3, yellow) superimposed onto the RET KD crystal structure (PDB ID code 2IVV, grey) showing no effect on the conformation of the kinase hinge by phospho-Y809.

(B) Schematic representation of basic residues engaged by either phospho-S909 and -Y928 from the JM-KD structure (PDB ID code 5FM2) or phospho-Y905 (PDB ID code 2IVV).

(C) WB analysis of in vitro time course autophosphorylation reaction using recombinant RET JM661-KD and RET KD WT and S909A mutants after addition of ATP (5 mM) and $MgCl_2$ (10 mM) for 0-80 minutes using the indicated antibodies.

(D) Enzymatic assay performed with recombinant purified (1µM) RET JM661-KD and RET KD WT and S909A mutants with increasing concentrations of ATP using an ABL derived peptide at a fixed concentration (4 mg/ml). Data represent mean ± SEM of n=3. Catalytic efficiency constants (k_{cat}/K_M, fold-difference) are shown on right panel.

(E) Phospho-S909 is not preferentially phosphorylated by RET catalytic domain in vitro. Enzyme kinetic experiment with recombinant purified phosphorylated-RET catalytic domain incubated with increasing concentrations of RET activation loop-derived peptides (with and without a phosphorylated S909 residue). Data shown are two independent experiments with different protein concentrations (5 µM left panel and 2.5 µM, right panel). These data is indicative of lack of intrinsic effect by phospho-S909 on RET catalytic activity.

(F) Quantitation of WB data of figure 4E. Data represent the mean of total autophosphorylation signal (percentage) ± SEM, n=3. Statistics *** $p<0.001$, ** $p< 0.01$, * $p< 0.05$, two- way Anova Bonferroni test versus control (JM-KD WT).

Figure S5 Plaza-Menacho et al.

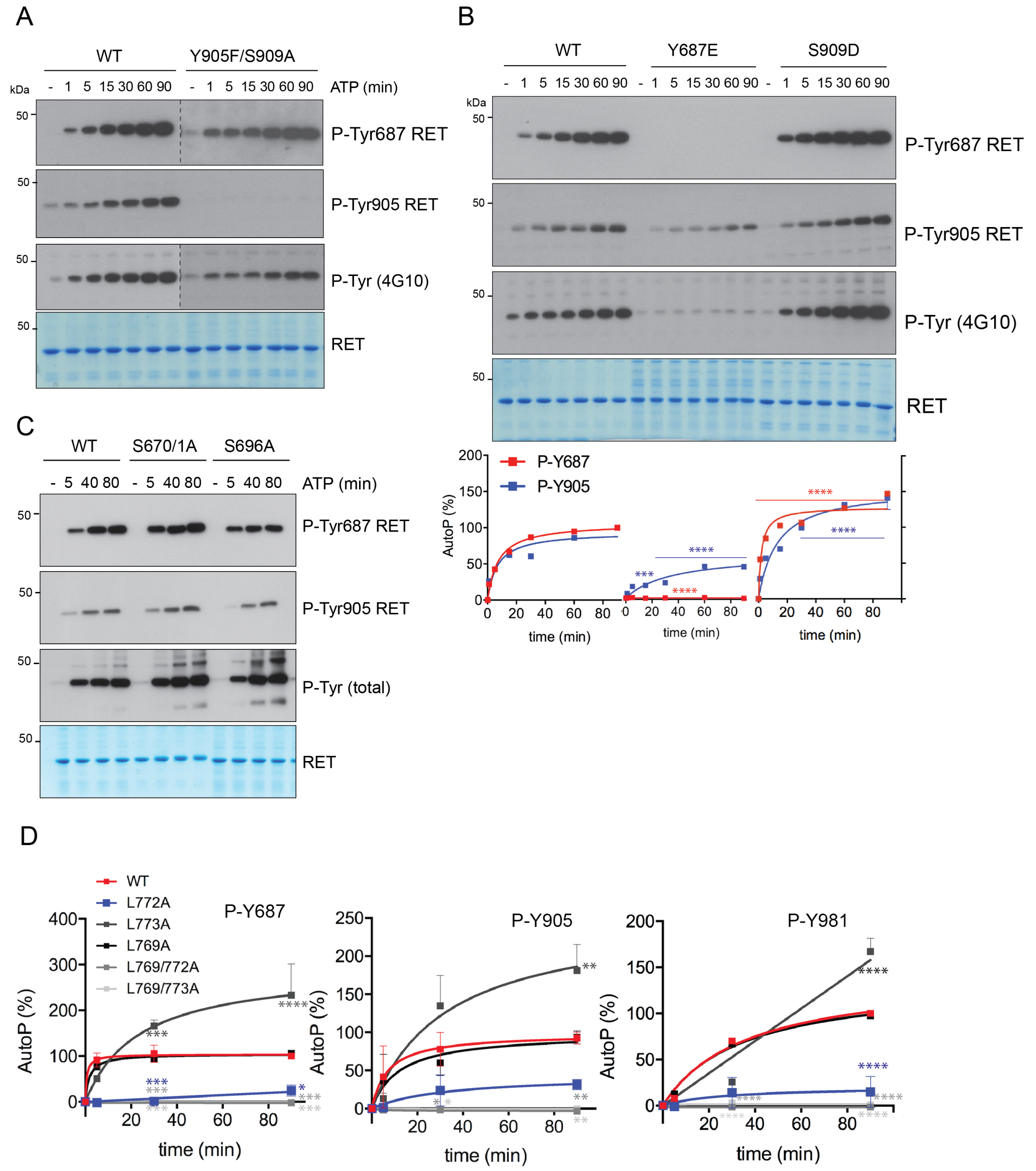

Figure S5 (related to figure 3, 4 and 5)

Functional characterization of distinct RET Tyr and Ser phospho-site mutants and quantitation of figure 5C autophosphorylation assay.

(A-C) WB analyses of recombinant RET JM-KD (1-2.5 µM) WT and indicated mutants incubated with saturating concentrations of ATP (5 mM) and $MgCl_2$ (10 mM) for 0-80/90 minutes using the indicated antibodies. Total amount of protein was assessed by Coomassie blue. Quantitation of WB data of figure S5B is depicted. Data represent the mean of autophosphorylation (percentage) ± SEM of the indicated antibodies, n=3. Statistics **** $p<0.0001$, *** $p<0.001$, two- way Anova Bonferroni test versus control (WT).

(D) Quantitation of WB data of figure 5C. Data represent the mean of autophosphorylation (percentage) ± SEM of the indicated antibodies, n=2. Statistics * $p< 0.05$, *** $p<0.001$, **** $p<0.0001$ two- way Anova Bonferroni test versus control (WT).

Figure S6   Plaza-Menacho et al.

A

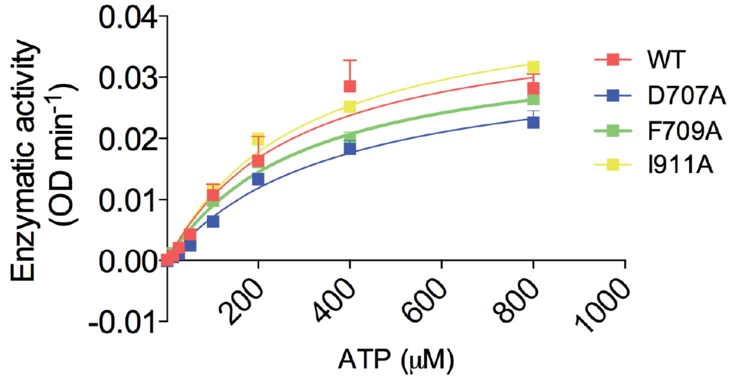

B

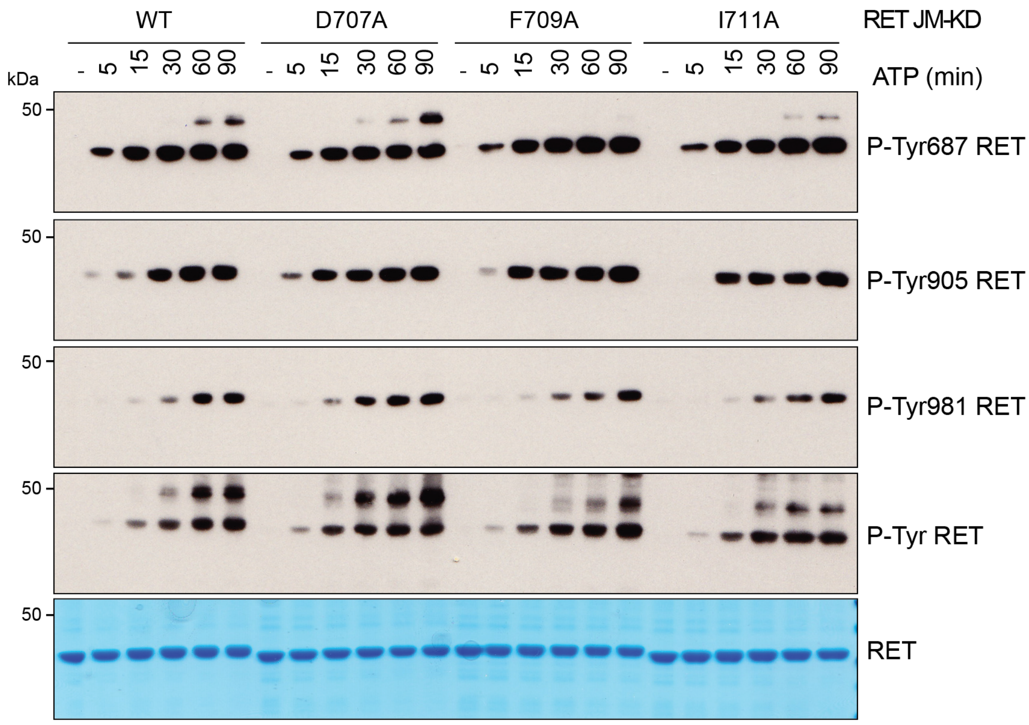

Figure S6 (related to figure 5 and 6)

Mutational analyses of RET DxFxI motif.

(A) Enzymatic assay performed with purified recombinant RET JM661-KD WT and indicated mutants (1 μM) incubated with increasing concentration of ATP using the ABL derived peptide EAIYAAPFAKKK at a constant 4 (mg/ml) concentration. Data represent mean ± SEM from 2 experiments with 6 replicates each, upper panel.

(B) WB analysis of recombinant purified RET samples from A (2.5 μM) stimulated with ATP (5 mM) and $MgCl_2$ (10 mM) for 0-80 minutes using the indicated antibodies, lower panel.
